# Gene expression changes in aging Zebrafish (*Danio rerio*) brains are sexually dimorphic

**DOI:** 10.1186/1471-2202-15-29

**Published:** 2014-02-18

**Authors:** Ayca Arslan-Ergul, Michelle M Adams

**Affiliations:** 1 BilGen Genetics and Biotechnology Center, Bilkent University, Ankara, Turkey; 2 Department of Molecular Biology and Genetics, Bilkent University, Ankara, Turkey; 3 Department of Psychology, Bilkent University 06800 Bilkent Ankara, Turkey

**Keywords:** Aging, Microarray, Brain, Gender, Synapse, Neurogenesis

## Abstract

**Background:**

Brain aging is a multi-factorial process due to both genetic and environmental factors. The zebrafish has recently become a popular model organism for examining aging and age-related diseases because as in humans they age gradually and exhibit cognitive decline. Few studies have examined the biological changes in the aging brain that may contribute to these declines and none have examined them within individuals with respect to gender. Our aim was to identify the main genetic pathways associated with zebrafish brain aging across gender. We chose males and females from specific age groups (young, 7.5-8.5 months and old, 31-36 months) based on the progression of cognitive decline in zebrafish. RNA was isolated from individual brains and subjected to microarray and qPCR analysis. Statistical analyses were performed using a two-way ANOVA and the relevant post-hoc tests.

**Results:**

Our results demonstrated that in the brains of young and old male and female zebrafish there were over 500 differentially expressed genes associated with multiple pathways but most notably were those related to neurogenesis and cell differentiation, as well as brain and nervous system development.

**Conclusions:**

The gene expression of multiple pathways is altered with age and differentially expressed in males and females. Future studies will be aimed at determining the causal relationships of age-related changes in gene expression in individual male and female brains, as well as possible interventions that counteract these alterations.

## Background

Both genetic and environmental factors contribute to brain aging and there is individual variation in these age-related changes. When working on processes that are associated with brain aging, gene expression profiling provides an efficient platform to observe the genetic activity at both an individual, and an organismal level. While many different model organisms have been used to examine the effects of aging, recently the zebrafish has become a popular model to study aging due to the ease with which genetic and environmental causes can be studied (reviewed in [1]). Zebrafish on average live about three years and age gradually like mammals [2]. They have an integrated nervous system and exhibit advanced behavioral properties like memory and social behaviors [3]. Moreover, it was demonstrated that aged zebrafish exhibit declines in spatial memory between 1 and 3 years [2,4]. In addition, senescence associated β-galactosidase (SA β-gal), which is a biomarker of aging, increases in a linear fashion with advancing age in zebrafish [5]. Thus, both behaviorally and biologically zebrafish show similar signs of aging as mammals.

The zebrafish genome is highly similar to the human genome and there are orthologs for 70% of the human genes [6]. However, currently there have not been any studies that systematically compared gene expression levels in individual young adult versus old male and female zebrafish brains. The existing studies either used undefined age groups, pooled samples, or ignored possible gender interactions. For example, one study examined the gene expression differences between mature male and female zebrafish, and the authors observed that forty-two genes were differentially expressed in individual brains between the sexes [7]. This study did not report the ages of the animals used but gave the body lengths and weights of the animals, which indicated that the fish were mature adults. Another study investigated the gene expression in the pineal glands and the brains of larval, 3 months old and 1-2 years old zebrafish [8], in which both the pineal gland samples and the brain samples were pooled and then analyzed. The results showed differential expression in larval and adult stages but reported no effects of gender. Microarray experiments have also been performed after starvation treatment in adult female zebrafish of 5-7 months of age [9], and only female fish were used in order to eliminate gender effects. In another study, the same group found that genes are differentially expressed in the zebrafish brain with respect to behaviorally distinct strains of fish [10]. Thus, genes are differentially regulated with respect to age and gender within individual subjects, and in order to determine the contributions to brain aging among individuals, one needs to understand the similarities and differences within subjects of specific ages and of different gender.

In this study, our aim was to identify the main genetic pathways that are associated with zebrafish brain aging that may contribute to cognitive decline. We chose specific age groups based on the progression of cognitive decline that occurs across the lifespan of the zebrafish and we utilized both male and female brains since it has been well-documented that like other mammals zebrafish brains are sexually dimorphic [11,12]. By using individual brains from males and females at specific ages and not pooled samples, we were able to determine whether there was variability among individuals. Our results demonstrated that over 500 genes were differentially expressed and multiple pathways are associated with these genes. The most notable were those related to neurogenesis and cell differentiation, as well as brain and nervous system development.

## Results

### Zebrafish exhibit age and gender related gene expression differences

**Figure 1:**
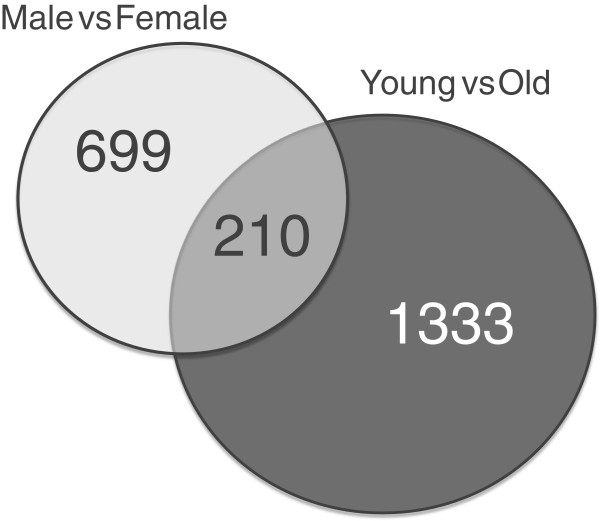
**A Venn diagram showing the number of differentially expressed transcripts.** There were 909 transcripts in Male vs Female and 1543 transcripts in Young vs Old comparisons. Two-hundred ten of differentially expressed transcripts were common to both lists.

A full list of 15,617 probesets with their corresponding p-values and Gene Ontology (GO) annotations are given in Additional file 1. In the same file, the probesets that were significant for female vs. male (FvsM), young vs. old (YvsO) and age by gender (AbyG) comparisons can be found. There were 909 differentially expressed transcripts in the female vs. male comparison, whereas 1543 transcripts were found in young vs. old comparison and 210 transcripts were common in both lists (Figure 1). The analysis of the age by gender interaction showed that 495 transcripts were significantly different (0.05 > p >?-0.05) (Additional file 1, Column H and Sheet 4). Thus, some genes are affected by age, gender and an age by gender dependency. At the time the lists were annotated, 2875 probesets out of 15,617 did not have a gene symbol but these were still associated with GO terms.

### Zebrafish transcripts are diversely enriched in Gene Ontology descriptions by age and gender

We classified the transcripts according to the GO descriptions. A graphical representation of GO terms distribution is shown in Additional file 2 for the female vs. male comparison and the young vs. old comparison. These figures show a general network of all the terms that are differentially expressed in either comparison and through these, interactions between the descriptions can be examined. We analyzed the list of all the GO terms that were significantly different in the two comparisons. Of particular interest to us, we found that for both the male versus female and old versus young comparisons, genes belonging to the following classifications were significantly changed; cell differentiation, neurogenesis, growth, cell proliferation, and development. In Table 1, the list of the selected GO terms is given. All of the descriptions that were listed were found to be significant (p < 0.05). The number of significant genes are given in the column X. Enrichment percentages are also given (Column E). All of the categories were enriched in male when compared for gender, and in young when compared for age. Thus, there are decreases in these genes in old animals compared to young and in females as compared to males. A full list of the GO terms can be found in Additional file 3 for both the female versus male and young versus old comparisons.

**Table 1:** Gene Ontology comparisons by gender and age

		**F vs. M comparison**		**Y vs. O comparison**	
**Description**	**GO ID**	**X**	**E**	**X**	**E**
Cellular component	5575	371	74% male	624	72% young
Biological process	8150	337	76% male	560	73% young
Cellular process	9987	176	77% male	336	75% young
Cell differentiation	30154	28	79% male	54	87% young
Regulation of gene expression	10468	31	68% male	49	80% young
Nervous system development	7399	35	83% male	45	86% young
Anatomical structure morphogenesis	9653	37	84% male	59	85% young
Neurogenesis	22008	18	78% male	30	83% young
Generation of neurons	48699	17	76% male	27	81% young
Angiogenesis	1525	10	90% male	12	75% young
Brain development	7420	13	69% male	18	94% young

All of the Gene Ontology Identification Numbers (GO IDs) listed here are significant (p < 0.05) in both young vs. old and female vs. male comparisons. *X*, Number of significant genes in the list; *E*, Enrichment.

### Eight genes were selected for microarray validations

We validated our microarray results by using quantitative real-time Polymerase Chain Reaction (qPCR). Before doing this we encountered two major limitations; first, not all the zebrafish probesets are annotated and secondly, we had a very small amount of RNA since we did not pool our samples. So we chose not to test large numbers of genes and selected a small set to validate the microarray results. We chose parvalbumin 8 (pvalb8), peroxisome proliferator-activated receptor gamma, coactivator 1 beta (ppargc1b), and insulin-like growth factor 2 mRNA binding protein 3 (igf2bp3) primarily because they were among the genes that exhibited the highest fold change. We selected insulin-like growth factor binding protein 2a (igfbp2a), insulin-like growth factor 1 (igf1), acetylcholinesterase (ache), SMAD specific E3 ubiquitin protein ligase 2 (smurf2), and LIM domain only 4a (lmo4a) because they were listed in GO terms that were significantly different in our comparisons. All of the selected genes for qPCR analysis have human and mouse homologues as listed in Table 2.

**Table 2:** Genes selected for qPCR validation

**Gene symbol (Zebrafish)**	**Gene name**	**Human ortholog**	**Mouse ortholog**
igf2bp3	Insulin-like growth factor 2 mRNA binding protein 3	IGF2BP3	Igf2bp3
ache	Acetylcholinesterase	ACHE	Ache
igf1	Insulin-like growth factor 1	IGF1	Igf1
pvalb8	parvalbumin 8	OCM2	Ocm
ppargc1b	peroxisome proliferator-activated receptor gamma, coactivator 1 beta	PPARGC1B	Ppargc1b
smurf2	SMAD specific E3 ubiquitin protein ligase 2	SMURF2	Smurf2
lmo4a	LIM domain only 4a	LMO4	Lmo4
igfbp2a	insulin-like growth factor binding protein 2a	IGFBP2	Igfbp2

In this table the gene name as well as the symbol for zebrafish and the human and mouse orthologues are listed.

From the microarray data, we generated a box-plot diagram showing the expression levels of the selected genes (Additional file 4). Here, we see that all of the genes exhibited a considerable amount of variation among samples; with the greatest variation observed in ppargc1b, and the lowest in igfbp2a. We also added beta-actin (bactin1) in this box-plot representation. It is interesting to note that while beta-actin had a very high expression level in all tissues, it did not show any variation; its expression remained stable among the samples. Because of this, we decided to take beta-actin as our reference gene for the qPCR experiments. In order to see the individual differences among the samples, we graphed each sample with their corresponding expression values (Additional file 5). Here, the samples are grouped into female-young (FY), female-old (FO), male-young (MY), and male-old (MO). While bactin was stable among and within groups, other genes showed differential variability. Ache, igfbp2a, pvalb8, and igf1 had higher expression in the MY group, whereas igf2bp3a, ppargc1b, and lmo4a favored both the MY and FY groups. Smurf2, on the other hand, had higher expressions in the MO group.

### qPCR results were in accordance with microarray results

Full statistical reports for both the microarray and qPCR analysis of the selected genes can be found in Additional file 6. This file is a series of SPSS report sheets of a two-way ANOVA test with both the F-values and corrected p-values, followed by univariate analysis and post-hoc tests.

**Figure 2:**
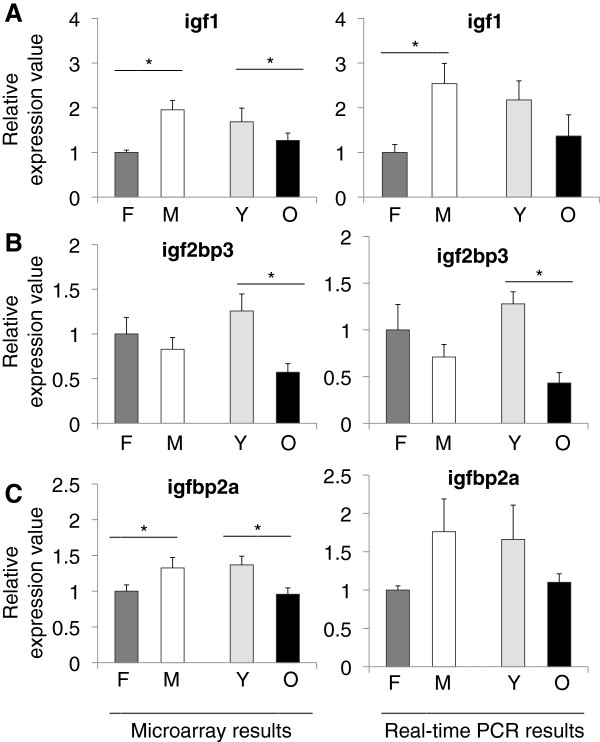
**Relative expression values from microarray and real-time PCR experiments for A) igf1, B) igf2bp3, and C) igfbp2a.** Values on the x-axis are the relative expressions in each group. In each graph the value for females are set to 1 as the reference for each group. F; female, M; male, Y; young, O; old. Error bars indicate the standard error, n =?6. *: p < 0.05.

Figure 2 shows the microarray and qPCR results for igf1, igf2bp3, and igfbp2a. Igf1 is a polypeptide hormone, which is synthesized mainly in liver but also in brain. It serves as a neurotrophic factor during development [13]. In general, the insulin/IGF1 pathways and related genes have been shown to have an effect on lifespan in model organisms [14] and variations in the genes of this pathway have been found to be associated with human lifespan [15,16]. Igf1 was affected significantly by age and gender in both the microarray (*F*_3, 8_ =?19.130, p =?0.01) and qPCR (*F*_3, 8_ =?5.096, p =?0.029) experiments. According to our microarray results, igf1 levels increased significantly in males when compared to females (p =?0.0001) and decreased in aged animals (p =?0.0324) (Figure 2A, left). Similarly, qPCR revealed a significant increase in males (p =?0.009) and a decrease in aged fish (Figure 2A, right). In addition, we analyzed the individual group differences in igf1 for both the microarray and qPCR data. Igf1 levels were highest in the young and old males as compared to the female groups (all p-values?≤?0.031, microarray; p =?0.037 (old females vs. young males), qPCR; Additional files 5 and 6).

Igf2bp3 (also known as IMP3 in human) binds to insulin-like growth factor 2 (igf2) mRNA on the 5′-Untranslated Region (UTR), thus regulating the expression of igf2, which is essential for fetal development [17]. Igf2bp3 was significantly associated with aging and gender according to the microarray (*F*_3, 8_ =?37.572, p < 0.005) and qPCR experimental results (*F*_3, 8_ =?18.055, p =?0.001). Igf2bp3 exhibited a significant decline with aging in the microarray and qPCR (p?≤?0.005) data (Figure 2B). When individual group differences of igf2bp3 levels were examined, we found that both young males and females had higher amounts of expression than old males and females (all p-values?≤?0.002, microarray; p-values?≤?0.001 (young females vs. old males and females), qPCR; Additional files 5 and 6). Similar results were obtained for igfbp2a, which showed an interaction in the microarray (*F*_3, 8_ =?6.176, p =?0.018) experiments. Igfbp2a is a secreted protein, which binds directly to igf. In zebrafish, the highest expression has been found to be in liver and the next highest level in the brain [18]. In our results, there was a decline with age (p =?0.009) and also in females relative to males (p =?0.034) (Figure 2C, left).

**Figure 3:**
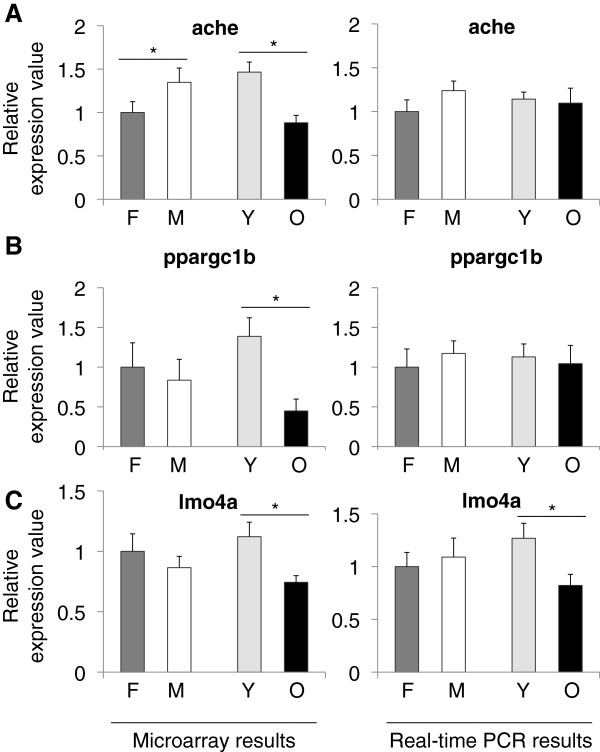
**Relative expression values from microarray and real-time PCR experiments for A) ache, B) ppargc1b, and C) lmo4a.** Values on the x-axis are the relative expressions in each group. In each graph the value for females are set to 1 as the reference for each group. F; female, M; male, Y; young, O; old. Error bars indicate the standard error, n =?6. *: p < 0.05.

The ache gene product, acetylcholinesterase (ACHE) is produced in the brain by cholinergic neurons. It is required for hydrolysis of acetylcholine and it has important roles in zebrafish development as revealed by experiments using ache mutants [19]. ACHE inhibitors are investigated largely as a treatment option for Alzheimer’s disease and an interaction was found between ache and presenilin-1 levels [20]. In our experiments, ache showed a decreased expression during aging (p =?0.0005) and an increased expression in males as compared to females (p =?0.0113) (Figure 3A). Moreover, in the microarray data we observed significant decreases in ache expression levels in old females and males as compared to young females and males (all p-values?≤?0.026; Additional files 5 and 6). Similarly, both ppargc1b (p =?0.0096) (Figure 3B) and lmo4a (p =?0.023) (Figure 3C) exhibited a significant decrease in aged animals. Ppargc1b is a coactivator of peroxisome proliferator activated receptor gamma (PPARγ), involved in lipid metabolism and regulates oxidative metabolism [21]. Lmo4a (LMO4 in human) is expressed in the telencephalon in zebrafish [22]. In humans, LMO4 is a suspected oncogene since it is found to be inhibiting the breast cancer type 1 susceptibility protein (BRCA1) activity [23]. In a thorough analysis of small nucleotide polymorphisms (SNPs) associated with human longevity and disease-free survival, LMO4 was listed as a near gene to a SNP locus [24].

**Figure 4:**
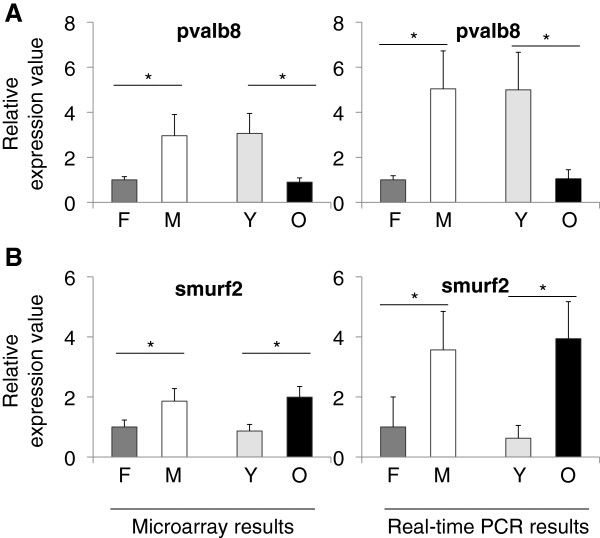
**Relative expression values from the microarray and real-time PCR experiments for A) pvalb8 and B) smurf2.** Values on the x-axis are the relative expressions in each group. In each graph the value for females are set to 1 as the reference for each group. F; female, M; male, Y; young, O; old. Error bars indicate the standard error, n =?6. *: p < 0.05.

Not much is known about the pvalb8 (also known as pvalb3a) protein. As a family, parvalbumins are calcium binding, low molecular weight proteins. Pvalb is expressed in GABAergic interneurons [25]. Pvalb8 was found to be orthologous to mammalian oncomodulin (OCM). In our data, dramatic changes were observed in pvalb8 expression levels (Figure 4A). Overall pvalb8 was significantly different in both the microarray (*F*_3, 8_ =?14.004, p =?0.002) and qPCR (*F*_3, 8_ =?52.133, p?≤?0.005) experiments. Specifically, there was a significant increase in males with respect to females (p =?0.008, microarray; p?≤?0.005, qPCR) and a significant decrease during aging (p =?0.001, microarray; p?≤?0.005, qPCR). More interestingly, pvalb8 expression exhibited a significant age by gender interaction (p =?0.027, microarray; p < 0.005, qPCR). In individual groups, young males had higher levels of pvalb8 expression than young females and old males and females (all p-values?≤?0.014, microarray; p =?0.0001 (old females vs. young males), qPCR; Additional files 5 and 6).

Smurf2 expression is increased during replicative senescence [26]. It has been shown that up-regulation of smurf2 alone without DNA damage is sufficient to drive fibroblasts into senescent state [26]. Replicative senescence is a consequence of progressive telomere shortening, which happens during the natural aging process in organisms. Smurf2, in our study, showed an overall significant change in both the microarray (*F*_3, 8_ =?5.746, p =?0.021) and in qPCR (*F*_3, 8_ =?7.302, p =?0.011) data (Figure 4B, left and right, respectively). There was an effect of gender (p =?0.048, microarray; p =?0.029, qPCR) and an increase during aging (p =?0.009, microarray; p =?0.009, qPCR). Moreover, an analysis of individual groups demonstrated a lower level in smurf2 expression levels in young males and females as compared to old males (p =?0.022 young females vs. old males, microarray; p-values?≤?0.038, qPCR; Additional files 5 and 6).

## Discussion

In this study, we examined the gene expression level changes that occur during normal aging, as well as the relationship with gender in zebrafish. For this we chose male and female fish at distinct ages; 7.5-8.5 months old fish for the young group and 31-36 month old fish for the old group. These ages were chosen because the cognitive changes that have been described in zebrafish correspond to these age groups [4]. Moreover, studies that have examined adult fish at distinct age groups are limited [7,8,10], and to our knowledge there are no studies of gene expression profiling of zebrafish brains in young and old, male and female adults at specific ages. Sexually dimorphic differences have been documented in the zebrafish brain [11,12]. In these studies, for example, the amount and pattern of cell proliferation in the brain is different between male and female zebrafish. To our knowledge, there are no studies that describe the gene expression differences with respect to an interaction with age and gender in the zebrafish brain. Another distinct advantage of our study was that we used individual brains, whereas other studies employed pooled brains due to small size of the sample. We overcame this problem by using RNA amplification steps prior to the microarray experiments and also by using only high quality RNA samples and highly efficient primers.

A study by Sreenivasan et al. [27] reported a sexually dimorphic pattern of gene expression in the zebrafish gonad and brain by using expressed sequence tags (ESTs). They generated a gonad-derived zebrafish cDNA microarray. They observed 24 candidate genes that showed a sexually dimorphic pattern but none of these genes were found among our list of genes that were affected by gender. This was probably due to the difference in starting material. In the Sreenivasan et al. study gonads were used as starting material and in our study we utilized brains. However, we did observe that the genes anti-Müllerian hormone (amh) and Wilms’ tumor suppressor gene 1 (wt1a) expression favored males and presenilin-1 (pre1) favored females in both studies.

### Various GO classifications are affected by gender and age in zebrafish

In the list of GO terms (Table 1), we chose general terms such as cellular component, biological process, cellular process, and terms related to development such as cell differentiation, regulation of gene expression, nervous system development. In all of these descriptions, it is very interesting to see that all categories were enriched in the males and in the young animals. This data would be in agreement with the findings from the Small et al. study [28], where they found a male-biased gene expression pattern. Although we only utilized adult tissues, aging can be considered as a form of adult development occurring at the end of life. For example, in the zebrafish brain, neurogenesis continues throughout adulthood and is a widespread process [29]. Thus, it was of particular interest to find the terms neurogenesis, angiogenesis and generation of neurons in our comparison lists because these processes are relevant to brain aging. In addition our data indicated that the genes associated with these processes are affected by gender with males having a higher level of expression of these genes. Similarly, processes that accompany neurogenesis such as regulation of gene expression, cell differentiation, and brain development were also found to be significant in both comparisons.

#### A subset of genes validates the microarray study and are affected by age and gender

We chose a small subset of genes to validate the microarray results and to have a closer look at the gene expression levels. In all three insulin-like growth factor (igf) pathway-related genes; igf1, igf2bp3, and igfbp2a, we observed a significant decline during aging (Figure 2). In terms of gender effects, we found a significant difference in igf1 and igfbp2a levels, with increased expression in males relative to females. Our findings are in support of a long-known fact that growth hormone levels [30] and specifically IGF1 levels [31] are lower in old animals. In addition to an age-related decline in igf1, we also observed a gender difference in the gene expression levels. We found that males had significantly more igf1 than females. This would be consistent with what has been described in rat brain [32].

In a study investigating glioblastoma survival, igf2bp3 was found to have gender association [33]. Hammer et al. detected the expression of igf2bp3 in the gonads with the testes having a higher level of expression than the ovary. This may explain the gender effect that we observed in our samples [34]. Interestingly, in a recent study, igf2bp3 was found to be among the list of genes that were found to have a lower expression in cirrhosis samples when compared to hepatocellular carcinoma tissues [35]. Cirrhosis is a senescent state of the liver. In our experiments we also observed a decrease in older animals. In accordance with our results, igfbp2a was shown to have high expression in male zebrafish when compared to females [36].

Figure 3 shows the expression values for three more genes of interest that we examined: ache, ppargc1b, and lmo4a. All three genes exhibited significant reductions during normal aging. In terms of gender, only ache showed a significant difference, favoring a higher expression in males. It is suggested that there is a reduction in cholinergic activity during normal aging [37], which may also explain the decrease in ache levels in our results. Changes in the expression levels of ache may contribute to cognitive changes. It has been shown in zebrafish that ache levels are related to cognitive performance during aging with an overexpression delaying the onset of cognitive decline [4]. Currently, experiments are ongoing in our laboratory aimed at examining the exact relationship between cholinergic activity, brain aging and gender.

Our analysis of ppargc1b and lmo4a showed significant decreases in aged zebrafish. Similar to our findings, ppargc1b mRNA levels were found to be decreased with age in a twin study investigating the susceptibility to type 2 diabetes [38]. To our knowledge, there are no studies that have investigated lmo4a and ppargc1b in zebrafish aging and it might be worth-while to study further.

Smurf2 exhibited dramatic increases during aging and also showed a sexually dimorphic pattern with an increase in males (Figure 4). This is very interesting because we know that smurf2 is implicated in replicative senescence. Smurf2 and its relationship with telomere-dependent senescence is also notable because although telomerase is active in older tissues in zebrafish, telomere lengths shorten as animals age, which has been shown in a comparison study between young and old fish [39]. Overall, it might be interesting to investigate further in zebrafish smurf2 and its role in aging.

Finally pvalb8 (oncomodulin in humans [40]) showed large decreases in old male as compared to young male zebrafish. A genome wide association study found a correlation between a SNP in the PVALB gene and female sexual dysfunction [41]. Pvalb8 expression was up regulated in zebrafish livers when exposed to 17alpha-ethynylestradiol (EE2) [42]. These findings correlate with our results showing a sexual-dimorphic pattern in pvalb8 expression. We observed a significant reduction in pvalb8 expression, which is consistent with an earlier study reporting reductions in parvalbumin expression in aging rat brain [43].

Overall the global direction of the changes that were observed in the microarray data was confirmed by the qPCR analysis. It was true that in a few cases while the direction of the changes was similar in both analyses, the data was not found to be significant. This is likely due to having more variability in the qPCR analyses. Moreover, in our current study we only utilized three animals per group in order to be able to study old and young male and female animals. While we were able to perform statistical analysis with three animals per group, it should be noted that in some cases it may be difficult to draw clear conclusions about no differences between groups due to the large amount of individual variation. Regardless of these discrepancies, we believe that the direction of changes that we have observed is significant to understanding the effects of the aging process on the male and female zebrafish brain.

## Conclusions

Our data suggest that the aging brain is undergoing many changes in gene expression and some of these alterations are related to gender. In order to understand the contributions of the neurobiological changes in the aging brain, one must examine both sexes. This is the first study that systematically examines age-related changes in male and female zebrafish brains. Future studies are ongoing to determine whether there are relationships between brain aging and behavior that vary across individual subjects. This is extremely important in considering possible interventions to alter the course of age-related declines and the zebrafish is an ideal model organism for testing possible drug or dietary interventions.

## Methods

### Animals

Four groups of wild-type zebrafish (AB strain) were used for this study. The distribution of the fish was young (7.5-8.5 months old) male and female and old (31-36 months old) male and female. There were three animals per group for a total of 12 animals in the study. All fish were raised in the zebrafish facility at Bilkent University, BilGen Genetics and Biotechnology Center, Ankara, Turkey. Fish were maintained at a constant temperature of 28°C on a 14:10hr light:dark cycle. All the fish were fed three times daily and kept in a recirculating system at all times. All animals were kept in groups, approximately 10 animals in a 4 L tank. Birthdates are recorded and only fish with the same birthdates are kept together in the same tank. For the brain dissection, fish were anesthetized in Tricaine (Sigma-Aldrich, Germany) and the head was separated from the body with a scalpel. The eyes/optic nerves were separated, and then the brain was dissected away from dorsal surface. Following visualization of the brain, it was removed from the skull. The tissues were weighed in 0.5 ml microtubes, frozen in liquid nitrogen and stored at -80**°**C until RNA isolation. During the brain dissections, internal organs including gonads were also dissected. In cases where there were eggs, the gender was determined as being female, and in cases where testes were found, the gender was determined as being male. Those animals with no visible eggs or testes were not included in the study. At the time of dissection, we weighed the dissected brains, which were 7.20?±?0.36 mg for male-young, 9.43?±?1.85 mg for male-old, 6.30?±?1.25 mg for female-young, and 8.47?±?0.45 mg for female-old.

The animal protocol for this study was approved by the Bilkent University Local Animal Ethics Committee (HADYEK) with the approval date: Feb 9, 2010 and no: 2010/1.

### RNA isolation

RNA isolation was performed with an RNAeasy Mini Kit (Qiagen, Germany). DNase treatment was done with RNase-free DNase Set (Qiagen, Germany). Each individual RNA sample was analyzed for RNA quality using an Agilent Bioanalyzer.

### Microarray

Microarray experiments were conducted in Almac Diagnostics, Craigavon, UK using the Affymetrix GeneChip Zebrafish Genome Array which covers 14,900 transcripts. Since we started with individual brains and not pooled samples, the RNA amount was not enough for microarray and downstream analysis, so the RNA was amplified using the Ovation RNA Amplification System V2 (NuGEN). The Encore® Biotin Module (NuGEN) was used to generate labeled cDNA products. The Raw data is available on Gene Expression Omnibus (GEO) with the accession number GSE53430.

### Quantitative real-time polymerase chain reaction (qPCR)

All qPCR experiments were done using the Roche LightCycler 480 System. All cDNAs were synthesized from a 500 ng RNA sample by using a Transcriptor First Strand cDNA Synthesis Kit (Roche, Germany). cDNA samples were all diluted to a 1:5 ratio and 3 μL were used for the following PCR experiments. Reactions were performed in 20 μL volume with Light cycler 480 SYBR Green I Master (Roche, Germany) and 1 μM of each primer. Primers were designed by using the Universal ProbeLibrary Assay Design Center specifically for zebrafish transcripts. Primer sequences and the corresponding PCR conditions can be found in Additional file 7. Each reaction was performed in duplicate, on separate plates. Relative quantification analysis was performed using LCS480 software (Roche, Germany).

### Microarray data analysis

Data analysis was done at AG Bioinformatics, Ankara, Turkey. Raw data was obtained from Almac Diagnostics, Craigavon, UK. Raw Affymetrix CEL files were processed with the RMA normalization method [44]. Data was analyzed in two directions; young vs. old, male vs. female, and a gender by age interaction. Probesets were annotated using BioMart (http://www.biomart.org/biomart/martview) with the current Zebrafish Genome Built (Danio rerio Zv9). Differentially expressed genes were identified using two-way between subjects ANOVA with a p-value less than 0.05. Transcripts showing significant changes (p < 0.05) in the ANOVA analysis were annotated with Gene Ontology (GO) terms [45]. The gene expression network was populated using Pearson correlation and shown in Cytoscape [46]. GO analysis was performed by the BiNGO plug-in (v. 2.44) of Cytoscape Software (v. 2.8.1) to list the over-represented transcripts (p < 0.05).

### qRT-PCR data analysis

2^(-ΔΔCt) method was employed to express fold changes. ΔCt was calculated as Ct (target gene) – Ct (reference gene). Actin was used as the reference gene. ΔΔCt was calculated as ΔCt of male/young/old - ΔCt of female, in order to calibrate against female values.

### Statistics

Statistical analysis for the selected genes with significant differences obtained from both the microarray data and the qPCR results were done using the SPSS program (IBM, Turkey). A two-way ANOVA test was employed to determine the significant differences between groups. Multivariate tests were performed to see significant interactions. Univariate tests were followed by post-hoc analysis with Tukey’s test and Bonferroni correction. Significance levels were set at p < 0.05.

## Competing interests

The authors declare that they have no competing interest.

## Authors’ contributions

AA-E participated in the microarray data analysis, performed the qPCR experiments and data analysis, and wrote the manuscript. MA participated in the study design, collected the brain tissues and coordinated and helped to finalize the manuscript. Both authors read and approved the final manuscript.

## Supplementary Material

Additional file 1 **In Sheet 1 (Data) 15,617 probes are given with their annotations obtained through BioMart (**http://www.biomart.org/biomart/martview**) with the current Zebrafish Genome Built (*Danio rerio*** **Zv9).** Probe Set Ids are found in column A, columns B and C display fold change ratios in log2 base of Female/Male and Young/Old, respectively. Columns D, E, and F show the corresponding p values for Female vs. Male, Young vs. Old, and Age by Gender interaction. In the following columns, annotation information and GO ontology information are given. Sheet 2 (FvsM) gives the list of 909 probesets that are significantly different between female and male, sheet 3 (YvsO) gives the list of 1543 probesets that are different between young and old. Sheet 4 (AbyG) shows the 495 probesets that have a significant difference in age by gender analysis. Intersection sheet shows the list of 210 genes that are common to FvsM and YvsO lists. Click here for file

Additional file 2 **Representative image of Go Analysis results.** Page 1; Female vs. Male, Page 2; Young vs. Old, Page 3; Color legend. In this picture colors more toward yellow mean relatively lower p-values, and colors more toward orange mean the highest p-values. Click here for file

Additional file 3 **A complete list of the Gene Ontology analysis.** For female vs. male comparison, 189 descriptions, their p-values, and the genes that were included in the description are given. For young vs. old comparison. 299 descriptions, their p-values, and the genes that were included in the description are given. Click here for file

Additional file 4 **Box plot representation of selected genes.** Expression values from microarray data in log2 base are given on y-axis. On the x-axis the genes are provided. Expression variation of all samples are summarized in one box for each gene. The horizontal lines in the boxes indicate the mean values. Actin values were the most stable among other genes. Click here for file

Additional file 5 **Individual expression values for samples from microarray analysis.** Expression values are actual linear values from microarray experiment. FO; female old, FY; female young, MO; male old, MY; male young. Expression values on Y-axis are displayed in units of hundreds (i.e. 80 represents 8000). Click here for file

Additional file 6 **SPSS statistical test report sheets for microarray and qPCR expressions of the selected genes.** On the first page, the first column indicates animal sex and age. The second and third columns indicate a number assignment as to the age or gender in order to perform the SPSS analysis. Columns four through eleven list the 2^-(target-actin) values for the PCR table, and log2-based expression values for the array table. Following sheets show the SPSS output files from multivariate analysis for the microarray and qPCR results, univariate analysis and the relevant post-hoc tests for the microarray and qPCR results, respectively. Click here for file

Additional file 7 **Genes selected for real-Time PCR experiments along with their corresponding RefSeq ID numbers used for microarray experiments and for primer design.** Primer sequences are given for each gene, left primer (top) and right primer (bottom). Amplicon lengths are given as nucleotide numbers and melting temperatures used for PCR are provided. UPL; universal probe library, Tm; melting temperature, nt; nucleotides. Click here for file
