# Supplementary figures and images for: Gene expression changes in aging Zebrafish (Danio rerio) brains are sexually dimorphic
Source: BMC Neurosci. 2014 Feb 18;15:29. doi: 10.1186/1471-2202-15-29 (PMC3937001; doi:10.1186/1471-2202-15-29)

GO Analysis representation of Female vs. Male

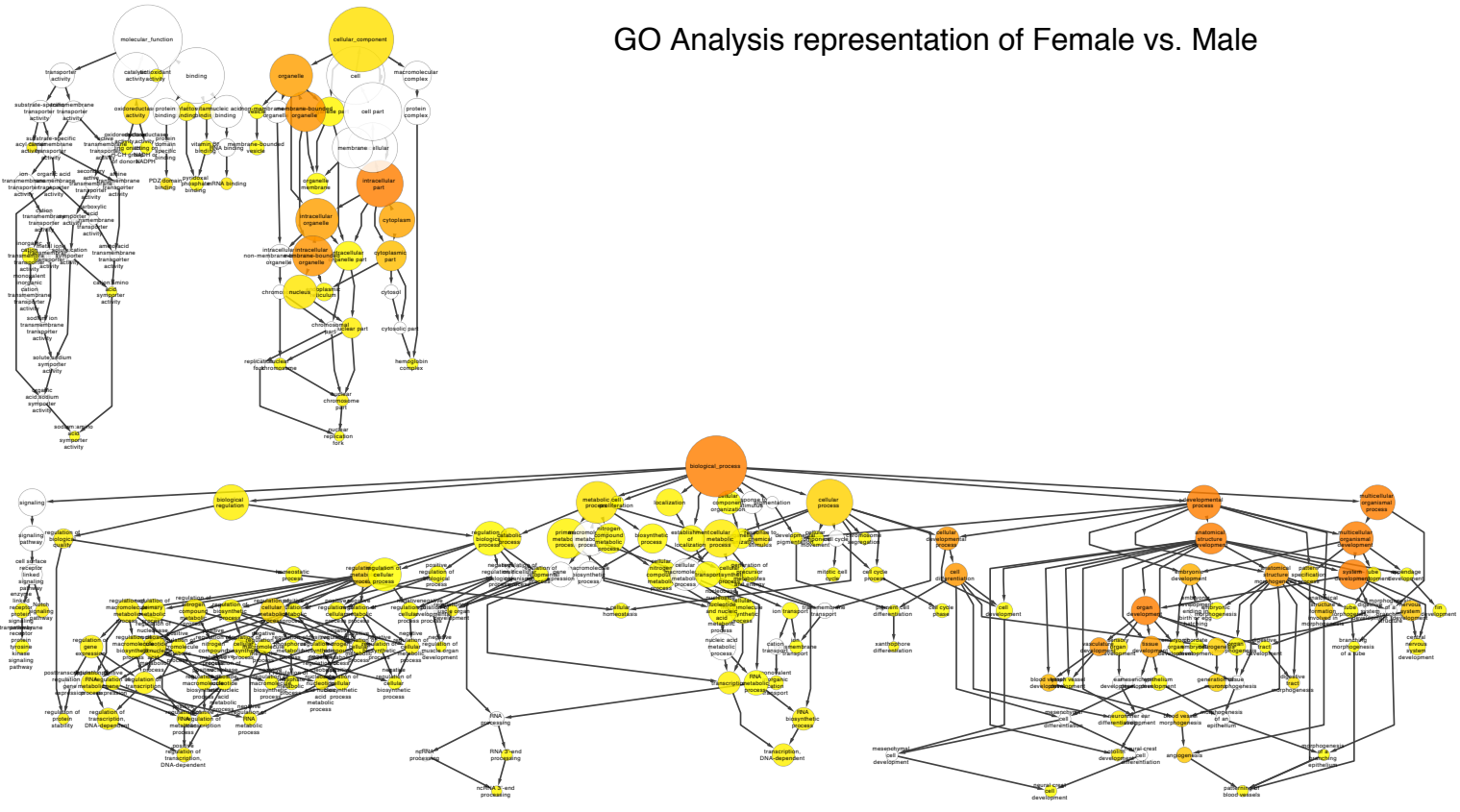

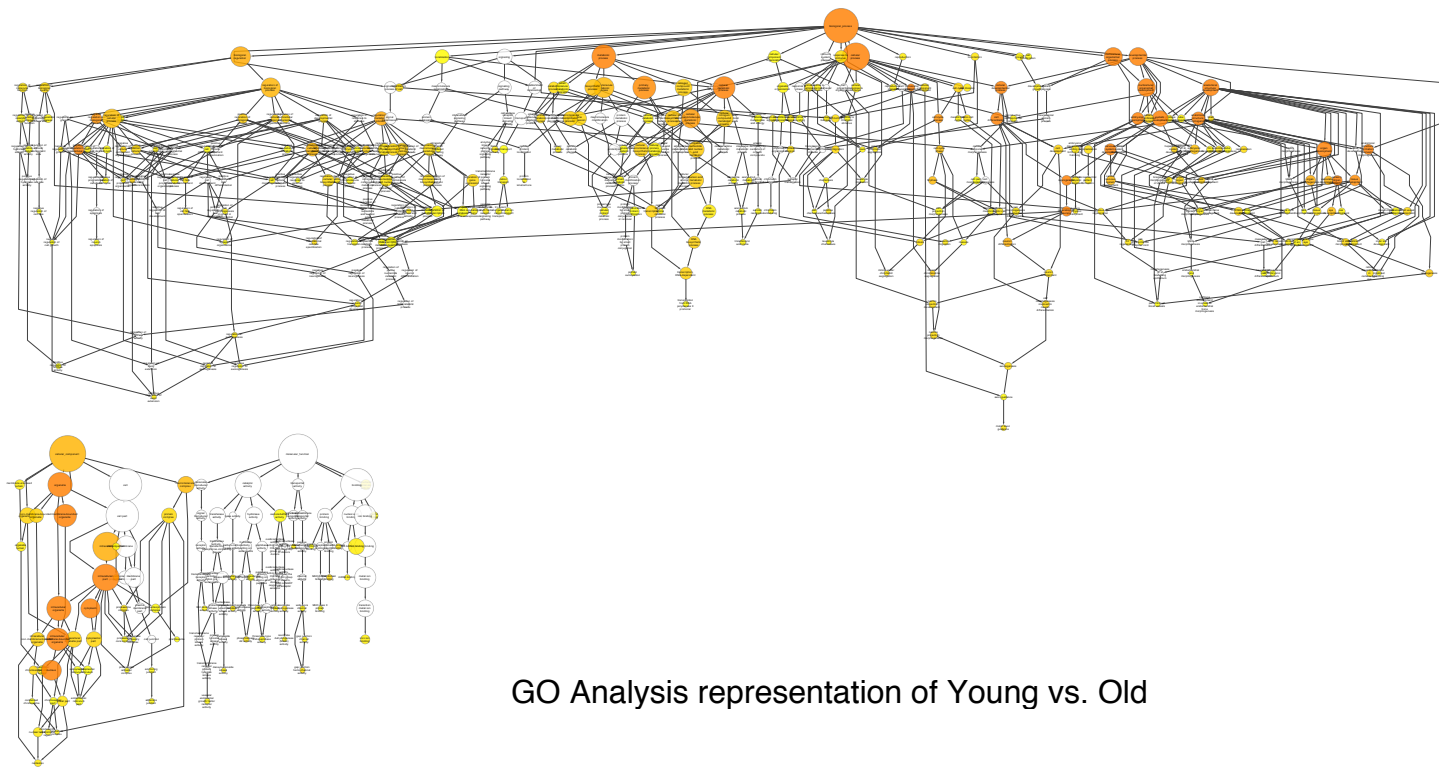

GO Analysis representation of Young vs. Old

5.00E-2

< 5.00E-7

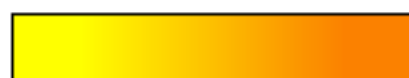

Supplement: Additional file 2 — Representative image of Go Analysis results. Page 1; Female vs. Male, Page 2; Young vs. Old, Page 3; Color legend. In this picture colors more toward yellow mean relatively lower p-values, and colors more toward orange mean the highest p-values. [file 1471-2202-15-29-S2.pdf]

## Additional File 4

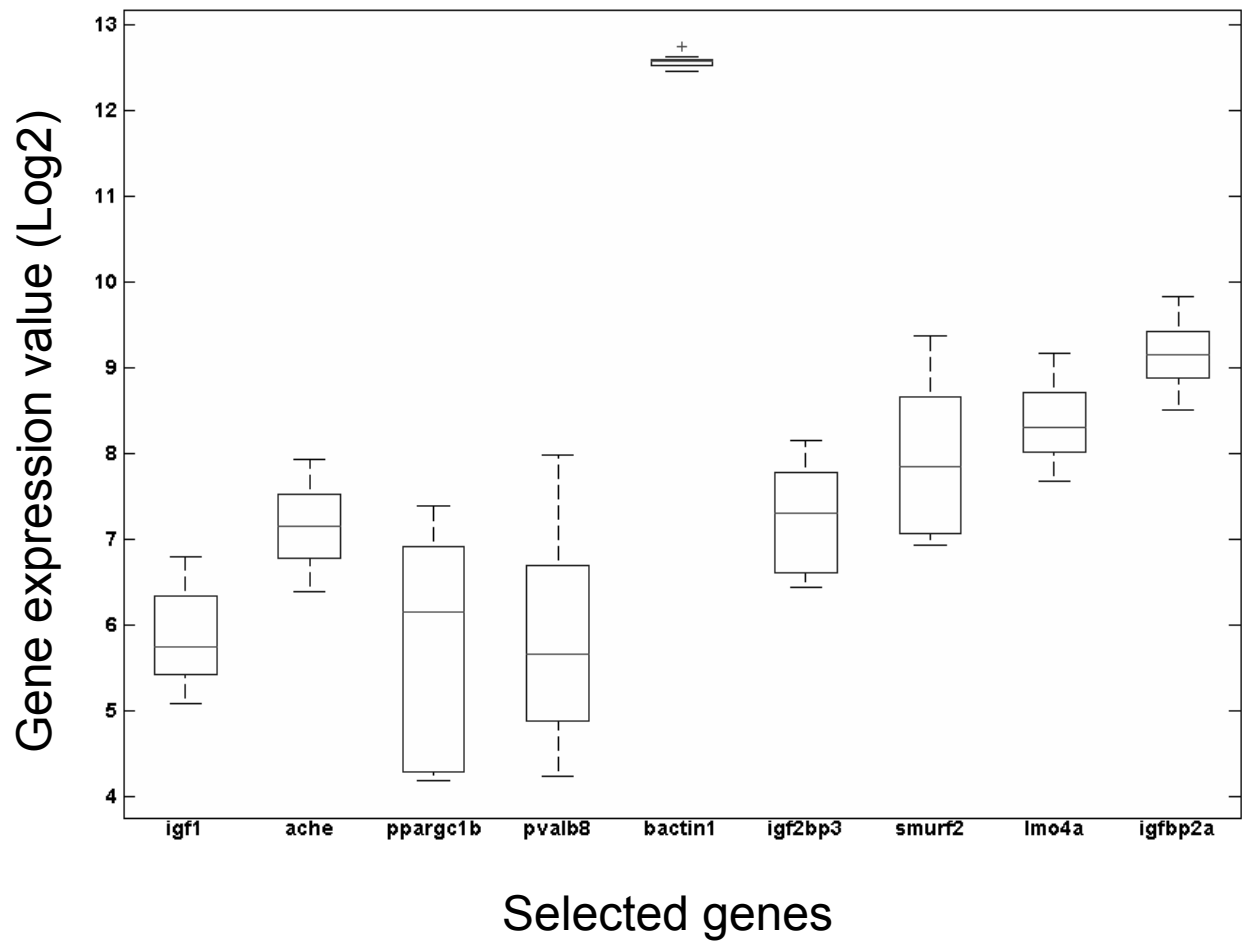

Supplement: Additional file 4 — Box plot representation of selected genes. Expression values from microarray data in log2 base are given on y-axis. On the x-axis the genes are provided. Expression variation of all samples are summarized in one box for each gene. The horizontal lines in the boxes indicate the mean values. Actin values were the most stable among other genes. [file 1471-2202-15-29-S4.pdf]

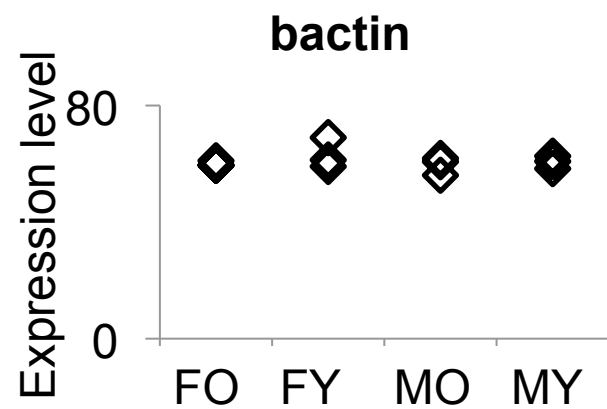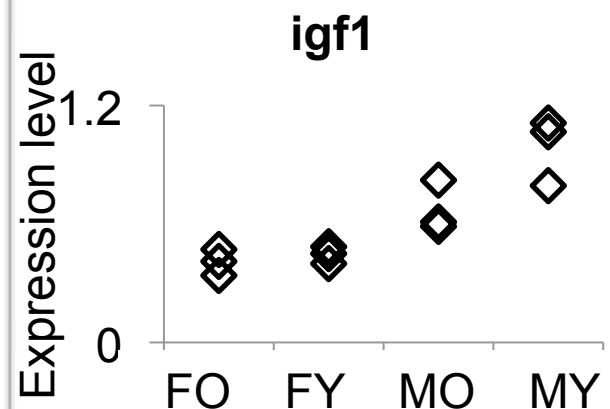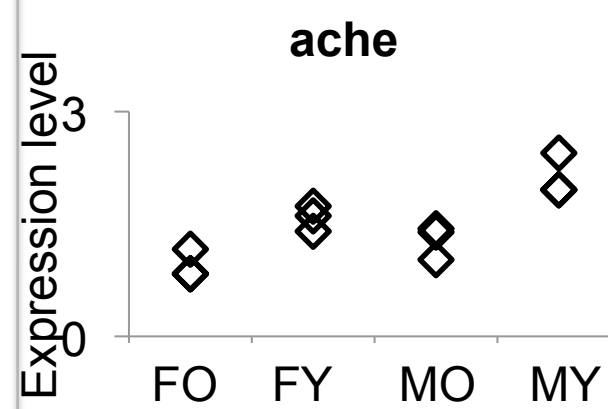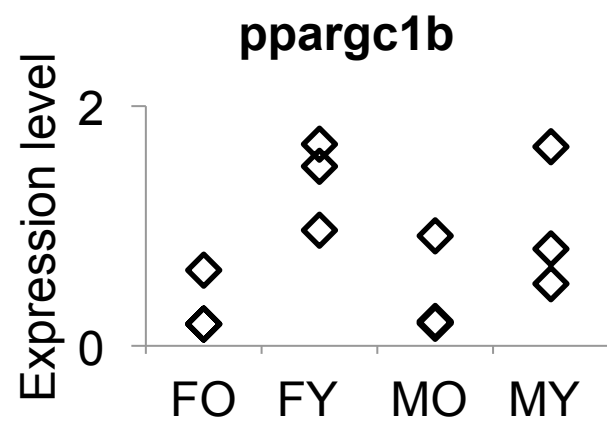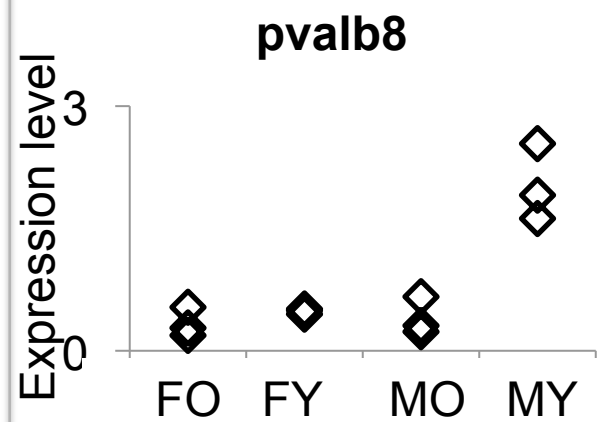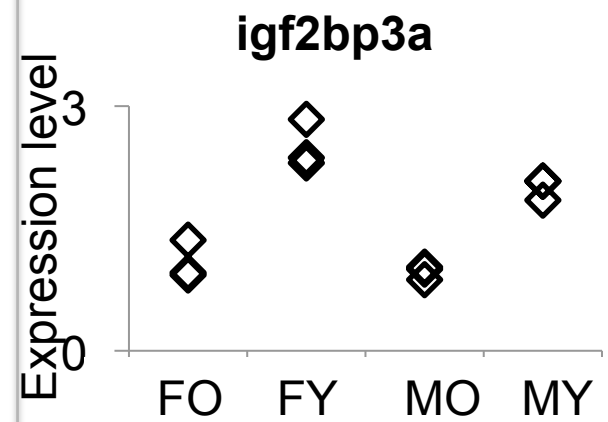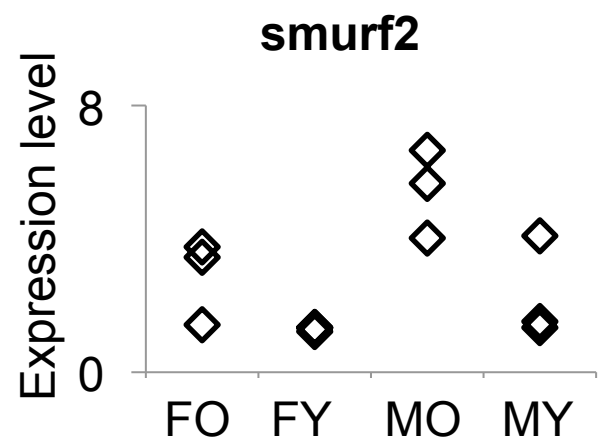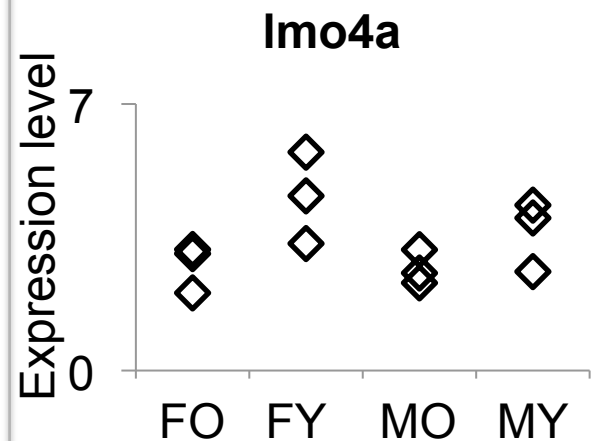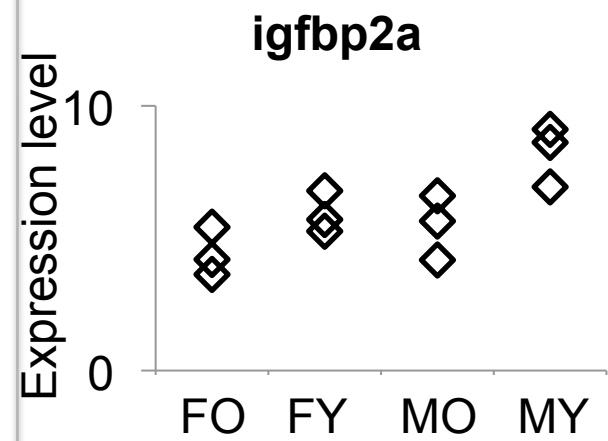

Supplement: Additional file 5 — Individual expression values for samples from microarray analysis. Expression values are actual linear values from microarray experiment. FO; female old, FY; female young, MO; male old, MY; male young. Expression values on Y-axis are displayed in units of hundreds (i.e. 80 represents 8000). [file 1471-2202-15-29-S5.pdf]
