# Supplementary material for: Gene expression changes in aging Zebrafish (Danio rerio) brains are sexually dimorphic
Source: BMC Neurosci. 2014 Feb 18;15:29. doi: 10.1186/1471-2202-15-29 (PMC3937001; doi:10.1186/1471-2202-15-29)
Supplement: Additional file 6 — SPSS statistical test report sheets for microarray and qPCR expressions of the selected genes. On the first page, the first column indicates animal sex and age. The second and third columns indicate a number assignment as to the age or gender in order to perform the SPSS analysis. Columns four through eleven list the 2^-(target-actin) values for the PCR table, and log2-based expression values for the array table. Following sheets show the SPSS output files from multivariate analysis for the microarray and qPCR results, univariate analysis and the relevant post-hoc tests for the microarray and qPCR results, respectively. [file 1471-2202-15-29-S6.pdf]

**PCR data**	gender	age	igf1	igf2bp3	igfbp2a	ache	ppargc1b	lmo4a	pvalb8	smurf2
male young	1	3	0.00143	0.000204	0.005805	0.00579	0.003035	0.00134	0.000539908	5.13242E-05
male young	1	3	0.00107	0.000200	0.00333	0.00652	0.00247	0.001004	0.00044931	6.5643E-05
male young	1	3	0.00129	0.000211	0.0114	0.006085	0.00342	0.00226	0.000580668	0.000809882
male old	1	4	0.00143	0.000123	0.00474	0.008825	0.005185	0.00097	4.9576E-05	0.001206464
male old	1	4	0.0007115	0.000067	0.002825	0.004685	0.00211	0.000803	0.000179342	0.001847769
male old	1	4	0.0002985	0.000071	0.003815	0.006075	0.004	0.001415	2.66593E-05	0.002306626
female young	2	3	0.0005765	0.000337	0.002715	0.00722	0.005285	0.001515	8.72189E-05	7.59284E-05
female young	2	3	0.0005035	0.000328	0.003185	0.004655	0.00191	0.00155	7.59284E-05	5.31342E-05
female young	2	3	0.0004625	0.000299	0.00365	0.004745	0.003345	0.0014	7.61921E-05	4.59365E-05
female old	2	4	0.000185	0.000039	0.00294	0.00549	0.00189	0.000991	4.59365E-05	4.73919E-05
female old	2	4	0.000179	0.000047	0.002485	0.00231	0.000879	0.000518	1.34224E-05	0.000220032
female old	2	4	0.0005455	0.000186	0.003145	0.006235	0.003935	0.001175	6.34069E-05	0.001320225

**array Data**	gender	age	igf1	igf2bp3	igfbp2a	ache	ppargc1b	lmo4a	pvalb8	smurf2
female old	2	4	5.359186	7.08402	8.504112	6.380872	4.179105	8.312779	5.737834	8.554103
female old	2	4	5.08436	6.581656	8.713191	6.387822	5.980089	7.671766	4.236365	8.429502
female old	2	4	5.557812	6.537725	9.081434	6.862578	4.19016	8.262496	4.80748	7.153806
female young	2	3	5.599444	7.887348	9.15605	7.329336	6.591848	9.163696	5.629529	6.932127
female young	2	3	5.49208	7.847375	9.407338	7.440911	7.226606	8.381575	5.488437	7.071434
female young	2	3	5.319539	8.14855	9.039871	7.131786	7.394426	8.841413	5.679785	7.056302
male old	1	4	6.361333	6.680798	9.14129	7.121068	4.231555	7.846972	6.050796	9.1456
male old	1	4	5.931561	6.645232	9.366686	6.67725	4.346971	8.000791	4.947777	8.652541
male old	1	4	5.877333	6.445622	8.706012	7.166621	6.520031	8.310153	4.545163	9.378325
male young	1	3	6.737187	7.529024	9.751043	7.934908	6.337207	8.762903	7.576195	7.064772
male young	1	3	6.796512	7.69888	9.831555	7.613174	7.376202	8.646268	7.9887	8.676047
male young	1	3	6.311233	7.702607	9.438425	7.61062	5.695138	8.021335	7.341379	7.249108

Microarray results - 8 genes statistics output from SPSS
Multivariate analysis

Descriptive Statistics

	gender	age	Mean	Std. Deviation	N
igf1	male	young	6.61497733	.264717451	3
		old	6.05674233	.265173103	3
		Total	6.33585983	.386839419	6
	female	young	5.47035433	.141211564	3
		old	5.33378600	.237745808	3
		Total	5.40207017	.190212468	6
	Total	young	6.04266583	.655022868	6
		old	5.69526417	.455560858	6
		Total	5.86896500	.567692060	12
igf2bp3	male	young	7.6435037	.09915981	3
		old	6.5905507	.12676543	3
		Total	7.1170272	.58563970	6
	female	young	7.9610910	.16356992	3
		old	6.7344670	.30351764	3
		Total	7.3477790	.70635201	6
	Total	young	7.8022973	.21188117	6
		old	6.6625088	.22246471	6
		Total	7.2324031	.63024341	12
igfbp2a	male	young	9.67367433	.207670971	3
		old	9.07132933	.335847294	3
		Total	9.37250183	.413780261	6
	female	young	9.20108633	.187827587	3
		old	8.76624567	.292294836	3
		Total	8.98366600	.324055722	6
	Total	young	9.43738033	.313630992	6
		old	8.91878750	.327436533	6
		Total	9.17808392	.408401651	12
ache	male	young	7.71956733	.186494860	3
		old	6.98831300	.270349610	3
		Total	7.35394017	.451184627	6
	female	young	7.30067767	.156542459	3
		old	6.54375733	.276128663	3
		Total	6.92221750	.460629514	6
	Total	young	7.51012250	.276323932	6
		old	6.76603517	.344997540	6
		Total	7.13807883	.489702040	12

Descriptive Statistics

	gender	age	Mean	Std. Deviation	N
ppargc1b	male	young	6.46951567	.848306095	3
		old	5.03285233	1.289226707	3
		Total	5.75118400	1.253751153	6
	female	young	7.07096000	.423322713	3
		old	4.78311800	1.036622031	3
		Total	5.92703900	1.439368350	6
	Total	young	6.77023783	.684142329	6
		old	4.90798517	1.055171297	6
		Total	5.83911150	1.290212842	12
lmo4a	male	young	8.47683533	.398762268	3
		old	8.05263867	.235903148	3
		Total	8.26473700	.373961897	6
	female	young	8.79556133	.393071363	3
		old	8.08234700	.356461307	3
		Total	8.43895417	.515005155	6
	Total	young	8.63619833	.394819523	6
		old	8.06749283	.270833452	6
		Total	8.35184558	.438638794	12
pvalb8	male	young	7.63542467	.327699915	3
		old	5.18124533	.779495529	3
		Total	6.40833500	1.446685545	6
	female	young	5.59925033	.099202382	3
		old	4.92722633	.757863232	3
		Total	5.26323833	.607588671	6
	Total	young	6.61733750	1.136086754	6
		old	5.05423583	.701530953	6
		Total	5.83578667	1.215208715	12
smurf2	male	young	7.66330900	.881886406	3
		old	9.05882200	.370591985	3
		Total	8.36106550	.974813716	6
	female	young	7.01995433	.076436083	3
		old	8.04580367	.775000794	3
		Total	7.53287900	.747193107	6
	Total	young	7.34163167	.661511913	6
		old	8.55231283	.776561461	6
		Total	7.94697225	.934221856	12

Tests of Between-Subjects Effects

Source	Dependent Variable	Type III Sum of Squares	df	Mean Square
Corrected Model	igf1	3.111[a]	3	1.037
	igf2bp3	4.080[b]	3	1.360
	igfbp2a	1.281[c]	3	.427
	ache	2.221[d]	3	.740
	ppargc1b	11.040[e]	3	3.680
	lmo4a	1.124[f]	3	.375
	pvalb8	13.646[g]	3	4.549
	smurf2	6.557[h]	3	2.186
Intercept	igf1	413.337	1	413.337
	igf2bp3	627.692	1	627.692
	igfbp2a	1010.847	1	1010.847
	ache	611.426	1	611.426
	ppargc1b	409.143	1	409.143
	lmo4a	837.040	1	837.040
	pvalb8	408.677	1	408.677
	smurf2	757.852	1	757.852
gender	igf1	2.616	1	2.616
	igf2bp3	.160	1	.160
	igfbp2a	.454	1	.454
	ache	.559	1	.559
	ppargc1b	.093	1	.093
	lmo4a	.091	1	.091
	pvalb8	3.934	1	3.934
	smurf2	2.058	1	2.058
age	igf1	.362	1	.362
	igf2bp3	3.897	1	3.897
	igfbp2a	.807	1	.807
	ache	1.661	1	1.661
	ppargc1b	10.404	1	10.404
	lmo4a	.970	1	.970
	pvalb8	7.330	1	7.330
	smurf2	4.397	1	4.397

Tests of Between-Subjects Effects

Source	Dependent Variable	F	Sig.
Corrected Model	igf1	19.130	.001
	igf2bp3	37.572	.000
	igfbp2a	6.176	.018
	ache	14.192	.001
	ppargc1b	4.049	.050
	lmo4a	3.020	.094
	pvalb8	14.004	.002
	smurf2	5.746	.021
Intercept	igf1	7624.179	.000
	igf2bp3	17341.923	.000
	igfbp2a	14616.265	.000
	ache	11723.146	.000
	ppargc1b	450.162	.000
	lmo4a	6747.174	.000
	pvalb8	1258.243	.000
	smurf2	1992.342	.000
gender	igf1	48.251	.000
	igf2bp3	4.413	.069
	igfbp2a	6.559	.034
	ache	10.721	.011
	ppargc1b	.102	.758
	lmo4a	.734	.417
	pvalb8	12.111	.008
	smurf2	5.409	.048
age	igf1	6.678	.032
	igf2bp3	107.676	.000
	igfbp2a	11.666	.009
	ache	31.847	.000
	ppargc1b	11.447	.010
	lmo4a	7.821	.023
	pvalb8	22.567	.001
	smurf2	11.560	.009

Tests of Between-Subjects Effects

Source	Dependent Variable	Type III Sum of Squares	df	Mean Square
gender * age	igf1	.133	1	.133
	igf2bp3	.023	1	.023
	igfbp2a	.021	1	.021
	ache	.000	1	.000
	ppargc1b	.543	1	.543
	lmo4a	.063	1	.063
	pvalb8	2.382	1	2.382
	smurf2	.102	1	.102
Error	igf1	.434	8	.054
	igf2bp3	.290	8	.036
	igfbp2a	.553	8	.069
	ache	.417	8	.052
	ppargc1b	7.271	8	.909
	lmo4a	.992	8	.124
	pvalb8	2.598	8	.325
	smurf2	3.043	8	.380
Total	igf1	416.882	12	
	igf2bp3	632.061	12	
	igfbp2a	1012.681	12	
	ache	614.064	12	
	ppargc1b	427.454	12	
	lmo4a	839.156	12	
	pvalb8	424.921	12	
	smurf2	767.453	12	
Corrected Total	igf1	3.545	11	
	igf2bp3	4.369	11	
	igfbp2a	1.835	11	
	ache	2.638	11	
	ppargc1b	18.311	11	
	lmo4a	2.116	11	
	pvalb8	16.244	11	
	smurf2	9.600	11	

Tests of Between-Subjects Effects

Source	Dependent Variable	F	Sig.
gender * age	igf1	2.460	.155
	igf2bp3	.625	.452
	igfbp2a	.304	.596
	ache	.009	.925
	ppargc1b	.598	.462
	lmo4a	.505	.498
	pvalb8	7.334	.027
	smurf2	.269	.618
Error	igf1		
	igf2bp3		
	igfbp2a		
	ache		
	ppargc1b		
	lmo4a		
	pvalb8		
	smurf2		
Total	igf1		
	igf2bp3		
	igfbp2a		
	ache		
	ppargc1b		
	lmo4a		
	pvalb8		
	smurf2		
Corrected Total	igf1		
	igf2bp3		
	igfbp2a		
	ache		
	ppargc1b		
	lmo4a		
	pvalb8		
	smurf2		

qPCR results - 8 genes statistics output from SPSS
Multivariate analysis

Descriptive Statistics

	age	gender	Mean	Std. Deviation	N
igf1	young	male	.0012633	.00018148	3
		female	.0005142	.00005774	3
		Total	.0008887	.00042765	6
	old	male	.0008133	.00057258	3
		female	.0003032	.00020989	3
		Total	.0005582	.00047628	6
	Total	male	.0010383	.00045284	6
		female	.0004087	.00017975	6
		Total	.0007235	.00046479	12
igf2bp3	young	male	.00020500	.000005568	3
		female	.00032100	.000020056	3
		Total	.00026300	.000064885	6
	old	male	.00008698	.000031254	3
		female	.00009042	.000082871	3
		Total	.00008870	.000056047	6
	Total	male	.00014599	.000067687	6
		female	.00020571	.000137326	6
		Total	.00017585	.000107829	12
igfbp2a	young	male	.00684500	.004134299	3
		female	.00318333	.000467502	3
		Total	.00501417	.003308585	6
	old	male	.00379333	.000957684	3
		female	.00285667	.000337799	3
		Total	.00332500	.000822016	6
	Total	male	.00531917	.003161904	6
		female	.00302000	.000406300	6
		Total	.00416958	.002461930	12
ache	young	male	.0061317	.00036723	3
		female	.0055400	.00145562	3
		Total	.0058358	.00100324	6
	old	male	.0065283	.00210690	3
		female	.0046783	.00208459	3
		Total	.0056033	.00213086	6
	Total	male	.0063300	.00136995	6
		female	.0051092	.00167585	6
		Total	.0057196	.00159252	12

Descriptive Statistics

	age	gender	Mean	Std. Deviation	N
ppargc1b	young	male	.00297500	.000477834	3
		female	.00351333	.001693785	3
		Total	.00324417	.001151449	6
	old	male	.00376500	.001550911	3
		female	.00223467	.001556882	3
		Total	.00299983	.001623040	6
	Total	male	.00337000	.001113863	6
		female	.00287400	.001614810	6
		Total	.00312200	.001347710	12
lmo4a	young	male	.0015347	.00065023	3
		female	.0014883	.00007848	3
		Total	.0015115	.00041501	6
	old	male	.0010627	.00031635	3
		female	.0008947	.00033893	3
		Total	.0009787	.00030732	6
	Total	male	.0012987	.00052535	6
		female	.0011915	.00039261	6
		Total	.0012451	.00044570	12
pvalb8	young	male	.00052329515	.00006723581	3
		female	.00007977979	.00000644378	3
		Total	.00030153747	.00024665083	6
	old	male	.00008519238	.00008233703	3
		female	.00004092193	.00002536677	3
		Total	.00006305715	.00005964148	6
	Total	male	.00030424376	.00024919917	6
		female	.00006035086	.00002696253	6
		Total	.00018229731	.00021161441	12
smurf2	young	male	.00030894986	.00043387934	3
		female	.00005833304	.00001565728	3
		Total	.00018364145	.00030698731	6
	old	male	.00178695288	.00055259702	3
		female	.00052921639	.00069045101	3
		Total	.00115808463	.00088735830	6
	Total	male	.00104795137	.00092346834	6
		female	.00029377471	.00050725382	6
		Total	.00067086304	.00081222748	12

Tests of Between-Subjects Effects

Source	Dependent Variable	Type III Sum of Squares	df	Mean Square
Corrected Model	igf1	1.560E-6[a]	3	5.200E-7
	igf2bp3	1.113E-7[b]	3	3.711E-8
	igfbp2a	2.999E-5[c]	3	9.996E-6
	ache	5.821E-6[d]	3	1.940E-6
	ppargc1b	4.127E-6[e]	3	1.376E-6
	lmo4a	8.973E-7[f]	3	2.991E-7
	pvalb8	4.686E-7[g]	3	1.562E-7
	smurf2	5.316E-6[h]	3	1.772E-6
Intercept	igf1	6.281E-6	1	6.281E-6
	igf2bp3	3.711E-7	1	3.711E-7
	igfbp2a	.000	1	.000
	ache	.000	1	.000
	ppargc1b	.000	1	.000
	lmo4a	1.860E-5	1	1.860E-5
	pvalb8	3.988E-7	1	3.988E-7
	smurf2	5.401E-6	1	5.401E-6
age	igf1	3.277E-7	1	3.277E-7
	igf2bp3	9.114E-8	1	9.114E-8
	igfbp2a	8.560E-6	1	8.560E-6
	ache	1.622E-7	1	1.622E-7
	ppargc1b	1.791E-7	1	1.791E-7
	lmo4a	8.517E-7	1	8.517E-7
	pvalb8	1.706E-7	1	1.706E-7
	smurf2	2.849E-6	1	2.849E-6
gender	igf1	1.189E-6	1	1.189E-6
	igf2bp3	1.070E-8	1	1.070E-8
	igfbp2a	1.586E-5	1	1.586E-5
	ache	4.471E-6	1	4.471E-6
	ppargc1b	7.380E-7	1	7.380E-7
	lmo4a	3.445E-8	1	3.445E-8
	pvalb8	1.785E-7	1	1.785E-7
	smurf2	1.706E-6	1	1.706E-6

Tests of Between-Subjects Effects

Source	Dependent Variable	F	Sig.
Corrected Model	igf1	5.096	.029
	igf2bp3	17.935	.001
	igfbp2a	2.180	.168
	ache	.703	.576
	ppargc1b	.694	.581
	lmo4a	1.858	.215
	pvalb8	52.134	.000
	smurf2	7.302	.011
Intercept	igf1	61.557	.000
	igf2bp3	179.317	.000
	igfbp2a	45.496	.000
	ache	142.256	.000
	ppargc1b	59.024	.000
	lmo4a	115.561	.000
	pvalb8	133.095	.000
	smurf2	22.258	.002
age	igf1	3.211	.111
	igf2bp3	44.043	.000
	igfbp2a	1.867	.209
	ache	.059	.815
	ppargc1b	.090	.771
	lmo4a	5.291	.050
	pvalb8	56.944	.000
	smurf2	11.740	.009
gender	igf1	11.656	.009
	igf2bp3	5.170	.053
	igfbp2a	3.458	.100
	ache	1.620	.239
	ppargc1b	.372	.559
	lmo4a	.214	.656
	pvalb8	59.558	.000
	smurf2	7.032	.029

Tests of Between-Subjects Effects

Source	Dependent Variable	Type III Sum of Squares	df	Mean Square
age * gender	igf1	4.284E-8	1	4.284E-8
	igf2bp3	9.503E-9	1	9.503E-9
	igfbp2a	5.569E-6	1	5.569E-6
	ache	1.188E-6	1	1.188E-6
	ppargc1b	3.210E-6	1	3.210E-6
	lmo4a	1.110E-8	1	1.110E-8
	pvalb8	1.195E-7	1	1.195E-7
	smurf2	7.607E-7	1	7.607E-7
Error	igf1	8.163E-7	8	1.020E-7
	igf2bp3	1.656E-8	8	2.069E-9
	igfbp2a	3.668E-5	8	4.586E-6
	ache	2.208E-5	8	2.760E-6
	ppargc1b	1.585E-5	8	1.982E-6
	lmo4a	1.288E-6	8	1.610E-7
	pvalb8	2.397E-8	8	2.996E-9
	smurf2	1.941E-6	8	2.426E-7
Total	igf1	8.658E-6	12	
	igf2bp3	4.990E-7	12	
	igfbp2a	.000	12	
	ache	.000	12	
	ppargc1b	.000	12	
	lmo4a	2.079E-5	12	
	pvalb8	8.914E-7	12	
	smurf2	1.266E-5	12	
Corrected Total	igf1	2.376E-6	11	
	igf2bp3	1.279E-7	11	
	igfbp2a	6.667E-5	11	
	ache	2.790E-5	11	
	ppargc1b	1.998E-5	11	
	lmo4a	2.185E-6	11	
	pvalb8	4.926E-7	11	
	smurf2	7.257E-6	11	

Tests of Between-Subjects Effects

Source	Dependent Variable	F	Sig.
age * gender	igf1	.420	.535
	igf2bp3	4.592	.064
	igfbp2a	1.215	.302
	ache	.430	.530
	ppargc1b	1.620	.239
	lmo4a	.069	.799
	pvalb8	39.899	.000
	smurf2	3.135	.115
Error	igf1		
	igf2bp3		
	igfbp2a		
	ache		
	ppargc1b		
	lmo4a		
	pvalb8		
	smurf2		
Total	igf1		
	igf2bp3		
	igfbp2a		
	ache		
	ppargc1b		
	lmo4a		
	pvalb8		
	smurf2		
Corrected Total	igf1		
	igf2bp3		
	igfbp2a		
	ache		
	ppargc1b		
	lmo4a		
	pvalb8		
	smurf2		

Univariate analysis with post-hoc tests
microarray results for the selected genes

```
SAVE OUTFILE='D:\Users\aycaergul-bcc\Desktop\array.sav'
 /COMPRESSED.
UNIANOVA igf1 BY array
  /METHOD=SSTYPE(3)
  /INTERCEPT=INCLUDE
  /POSTHOC=array(TUKEY BONFERRONI)
  /CRITERIA=ALPHA(0.05)
  /DESIGN=array.
```

Univariate Analysis of Variance

Notes

Output Created		20-Ara-2013 19:24:53
Comments		
Input	Data	D:\Users\aycaergul-bcc\Desktop\array.sav
	Active Dataset	DataSet1
	Filter	<none>
	Weight	<none>
	Split File	<none>
	N of Rows in Working Data File	12
Missing Value Handling	Definition of Missing	User-defined missing values are treated as missing.
	Cases Used	Statistics are based on all cases with valid data for all variables in the model.
Syntax		UNIANOVA igf1 BY array /METHOD=SSTYPE(3) /INTERCEPT=INCLUDE /POSTHOC=array(TUKEY BONFERRONI) /CRITERIA=ALPHA(0.05) /DESIGN=array.
Resources	Processor Time	00 00:00:00,031
	Elapsed Time	00 00:00:00,031

```
[DataSet1] D:\Users\aycaergul-bcc\Desktop\array.sav
```

Between-Subjects Factors

		Value Label	N
array	1	female old	3
	2	female young	3
	3	male old	3
	4	male young	3

Tests of Between-Subjects Effects

Dependent Variable:igf1

Source	Type III Sum of Squares	df	Mean Square	F	Sig.
Corrected Model	3,111[a]	3	1,037	19,130	,001
Intercept	413,337	1	413,337	7624,179	,000
array	3,111	3	1,037	19,130	,001
Error	,434	8	,054		
Total	416,882	12			
Corrected Total	3,545	11			

a. R Squared = ,878 (Adjusted R Squared = ,832)

Post Hoc Tests

array

Multiple Comparisons

Dependent Variable:igf1

	(I) array	(J) array	Mean Difference (I-J)	Std. Error	Sig.
Tukey HSD	female old	female young	-,136568	,1901122	,887
		male old	-,722956*	,1901122	,022
		male young	-1,281191*	,1901122	,001
	female young	female old	,136568	,1901122	,887
		male old	-,586388	,1901122	,059
		male young	-1,144623*	,1901122	,001
	male old	female old	,722956*	,1901122	,022
		female young	,586388	,1901122	,059
		male young	-,558235	,1901122	,073
	male young	female old	1,281191*	,1901122	,001
		female young	1,144623*	,1901122	,001
		male old	,558235	,1901122	,073
Bonferroni	female old	female young	-,136568	,1901122	1,000
		male old	-,722956*	,1901122	,031
		male young	-1,281191*	,1901122	,001
	female young	female old	,136568	,1901122	1,000
		male old	-,586388	,1901122	,090
		male young	-1,144623*	,1901122	,002
	male old	female old	,722956*	,1901122	,031
		female young	,586388	,1901122	,090
		male young	-,558235	,1901122	,113
	male young	female old	1,281191*	,1901122	,001
		female young	1,144623*	,1901122	,002
		male old	,558235	,1901122	,113

Multiple Comparisons

Dependent Variable:igf1

	(I) array	(J) array	95% Confidence Interval Lower Bound	95% Confidence Interval Upper Bound
Tukey HSD	female old	female young	-,745374	,472238
		male old	-1,331762	-,114150
		male young	-1,889997	-,672385
	female young	female old	-,472238	,745374
		male old	-1,195194	,022418
		male young	-1,753429	-,535817
	male old	female old	,114150	1,331762
		female young	-,022418	1,195194
		male young	-1,167041	,050571
	male young	female old	,672385	1,889997
		female young	,535817	1,753429
		male old	-,050571	1,167041
Bonferroni	female old	female young	-,797946	,524809
		male old	-1,384334	-,061579
		male young	-1,942569	-,619814
	female young	female old	-,524809	,797946
		male old	-1,247765	,074989
		male young	-1,806000	-,483246
	male old	female old	,061579	1,384334
		female young	-,074989	1,247765
		male young	-1,219612	,103142
	male young	female old	,619814	1,942569
		female young	,483246	1,806000
		male old	-,103142	1,219612

Based on observed means.
The error term is Mean Square(Error) = ,054.

*. The mean difference is significant at the 0,05 level.

Homogeneous Subsets

igf1

	array	N	Subset 1	Subset 2	Subset 3
Tukey HSD[a,b]	female old	3	5,333786		
	female young	3	5,470354	5,470354	
	male old	3		6,056742	6,056742
	male young	3			6,614977
	Sig.		,887	,059	,073

Means for groups in homogeneous subsets are displayed.
Based on observed means.
The error term is Mean Square(Error) = ,054.

a. Uses Harmonic Mean Sample Size = 3,000.
b. Alpha = 0,05.

```
UNIANOVA igf2bp3 BY array
  /METHOD=SSTYPE(3)
  /INTERCEPT=INCLUDE
  /POSTHOC=array(TUKEY BONFERRONI)
  /CRITERIA=ALPHA(0.05)
  /DESIGN=array.
```

Univariate Analysis of Variance

Notes

Output Created		20-Ara-2013 19:25:16
Comments		
Input	Data	D:\Users\aycaergul-bcc\Desktop\array.sav
	Active Dataset	DataSet1
	Filter	<none>
	Weight	<none>
	Split File	<none>
	N of Rows in Working Data File	12
Missing Value Handling	Definition of Missing	User-defined missing values are treated as missing.
	Cases Used	Statistics are based on all cases with valid data for all variables in the model.
Syntax		UNIANOVA igf2bp3 BY array /METHOD=SSTYPE(3) /INTERCEPT=INCLUDE /POSTHOC=array(TUKEY BONFERRONI) /CRITERIA=ALPHA(0.05) /DESIGN=array.
Resources	Processor Time	00 00:00:00,000
	Elapsed Time	00 00:00:00,000

```
[DataSet1] D:\Users\aycaergul-bcc\Desktop\array.sav
```

Between-Subjects Factors

		Value Label	N
array	1	female old	3
	2	female young	3
	3	male old	3
	4	male young	3

Tests of Between-Subjects Effects

Dependent Variable:igf2bp3

Source	Type III Sum of Squares	df	Mean Square	F	Sig.
Corrected Model	4,080[a]	3	1,360	37,572	,000
Intercept	627,692	1	627,692	17341,923	,000
array	4,080	3	1,360	37,572	,000
Error	,290	8	,036		
Total	632,061	12			
Corrected Total	4,369	11			

a. R Squared = ,934 (Adjusted R Squared = ,909)

Post Hoc Tests

array

Multiple Comparisons

Dependent Variable:igf2bp3

	(I) array	(J) array	Mean Difference (I-J)	Std. Error	Sig.
Tukey HSD	female old	female young	-1,226624*	,1553385	,000
		male old	,143916	,1553385	,792
		male young	-,909037*	,1553385	,002
	female young	female old	1,226624*	,1553385	,000
		male old	1,370540*	,1553385	,000
		male young	,317587	,1553385	,249
	male old	female old	-,143916	,1553385	,792
		female young	-1,370540*	,1553385	,000
		male young	-1,052953*	,1553385	,001
	male young	female old	,909037*	,1553385	,002
		female young	-,317587	,1553385	,249
		male old	1,052953*	,1553385	,001
Bonferroni	female old	female young	-1,226624*	,1553385	,000
		male old	,143916	,1553385	1,000
		male young	-,909037*	,1553385	,002
	female young	female old	1,226624*	,1553385	,000
		male old	1,370540*	,1553385	,000
		male young	,317587	,1553385	,451
	male old	female old	-,143916	,1553385	1,000
		female young	-1,370540*	,1553385	,000
		male young	-1,052953*	,1553385	,001
	male young	female old	,909037*	,1553385	,002
		female young	-,317587	,1553385	,451
		male old	1,052953*	,1553385	,001

Multiple Comparisons

Dependent Variable:igf2bp3

	(I) array	(J) array	95% Confidence Interval Lower Bound	95% Confidence Interval Upper Bound
Tukey HSD	female old	female young	-1,724072	-,729176
		male old	-,353532	,641365
		male young	-1,406485	-,411588
	female young	female old	,729176	1,724072
		male old	,873092	1,867989
		male young	-,179861	,815036
	male old	female old	-,641365	,353532
		female young	-1,867989	-,873092
		male young	-1,550401	-,555505
	male young	female old	,411588	1,406485
		female young	-,815036	,179861
		male old	,555505	1,550401
Bonferroni	female old	female young	-1,767028	-,686220
		male old	-,396487	,684320
		male young	-1,449440	-,368633
	female young	female old	,686220	1,767028
		male old	,830137	1,910944
		male young	-,222816	,857991
	male old	female old	-,684320	,396487
		female young	-1,910944	-,830137
		male young	-1,593357	-,512549
	male young	female old	,368633	1,449440
		female young	-,857991	,222816
		male old	,512549	1,593357

Based on observed means.
The error term is Mean Square(Error) = ,036.

*. The mean difference is significant at the 0,05 level.

Homogeneous Subsets

igf2bp3

	array	N	Subset	
			1	2
Tukey HSD[a,b]	male old	3	6,590551	
	female old	3	6,734467	
	male young	3		7,643504
	female young	3		7,961091
	Sig.		,792	,249

Means for groups in homogeneous subsets are displayed.
Based on observed means.
The error term is Mean Square(Error) = ,036.

a. Uses Harmonic Mean Sample Size = 3,000.
b. Alpha = 0,05.

```
UNIANOVA igfbp2a BY array
  /METHOD=SSTYPE(3)
  /INTERCEPT=INCLUDE
  /POSTHOC=array(TUKEY BONFERRONI)
  /CRITERIA=ALPHA(0.05)
  /DESIGN=array.
```

Univariate Analysis of Variance

Notes

Output Created		20-Ara-2013 19:25:32
Comments		
Input	Data	D:\Users\aycaergul-bcc\Desktop\array.sav
	Active Dataset	DataSet1
	Filter	<none>
	Weight	<none>
	Split File	<none>
	N of Rows in Working Data File	12
Missing Value Handling	Definition of Missing	User-defined missing values are treated as missing.
	Cases Used	Statistics are based on all cases with valid data for all variables in the model.
Syntax		UNIANOVA igfbp2a BY array /METHOD=SSTYPE(3) /INTERCEPT=INCLUDE /POSTHOC=array(TUKEY BONFERRONI) /CRITERIA=ALPHA(0.05) /DESIGN=array.
Resources	Processor Time	00 00:00:00,000
	Elapsed Time	00 00:00:00,000

```
[DataSet1] D:\Users\aycaergul-bcc\Desktop\array.sav
```

Between-Subjects Factors

		Value Label	N
array	1	female old	3
	2	female young	3
	3	male old	3
	4	male young	3

Tests of Between-Subjects Effects

Dependent Variable:igfbp2a

Source	Type III Sum of Squares	df	Mean Square	F	Sig.
Corrected Model	1,281[a]	3	,427	6,176	,018
Intercept	1010,847	1	1010,847	14616,265	,000
array	1,281	3	,427	6,176	,018
Error	,553	8	,069		
Total	1012,681	12			
Corrected Total	1,835	11			

a. R Squared = ,698 (Adjusted R Squared = ,585)

Post Hoc Tests

array

Multiple Comparisons

Dependent Variable:igfbp2a

	(I) array	(J) array	Mean Difference (I-J)	Std. Error	Sig.
Tukey HSD	female old	female young	-,434841	,2147231	,256
		male old	-,305084	,2147231	,522
		male young	-,907429*	,2147231	,012
	female young	female old	,434841	,2147231	,256
		male old	,129757	,2147231	,928
		male young	-,472588	,2147231	,203
	male old	female old	,305084	,2147231	,522
		female young	-,129757	,2147231	,928
		male young	-,602345	,2147231	,088
	male young	female old	,907429*	,2147231	,012
		female young	,472588	,2147231	,203
		male old	,602345	,2147231	,088
Bonferroni	female old	female young	-,434841	,2147231	,465
		male old	-,305084	,2147231	1,000
		male young	-,907429*	,2147231	,017
	female young	female old	,434841	,2147231	,465
		male old	,129757	,2147231	1,000
		male young	-,472588	,2147231	,353
	male old	female old	,305084	,2147231	1,000
		female young	-,129757	,2147231	1,000
		male young	-,602345	,2147231	,138
	male young	female old	,907429*	,2147231	,017
		female young	,472588	,2147231	,353
		male old	,602345	,2147231	,138

Multiple Comparisons

Dependent Variable:igfbp2a

	(I) array	(J) array	95% Confidence Interval Lower Bound	Upper Bound
Tukey HSD	female old	female young	-1,122460	,252778
		male old	-,992703	,382535
		male young	-1,595048	-,219810
	female young	female old	-,252778	1,122460
		male old	-,557862	,817376
		male young	-1,160207	,215031
	male old	female old	-,382535	,992703
		female young	-,817376	,557862
		male young	-1,289964	,085274
	male young	female old	,219810	1,595048
		female young	-,215031	1,160207
		male old	-,085274	1,289964
Bonferroni	female old	female young	-1,181836	,312155
		male old	-1,052079	,441912
		male young	-1,654424	-,160433
	female young	female old	-,312155	1,181836
		male old	-,617239	,876753
		male young	-1,219584	,274408
	male old	female old	-,441912	1,052079
		female young	-,876753	,617239
		male young	-1,349341	,144651
	male young	female old	,160433	1,654424
		female young	-,274408	1,219584
		male old	-,144651	1,349341

Based on observed means.
The error term is Mean Square(Error) = ,069.

*. The mean difference is significant at the 0,05 level.

Homogeneous Subsets

igfbp2a

	array	N	Subset 1	Subset 2
Tukey HSD[a,b]	female old	3	8,766246	
	male old	3	9,071329	9,071329
	female young	3	9,201086	9,201086
	male young	3		9,673674
	Sig.		,256	,088

Means for groups in homogeneous subsets are displayed.
Based on observed means.
The error term is Mean Square(Error) = ,069.

a. Uses Harmonic Mean Sample Size = 3,000.
b. Alpha = 0,05.

```
UNIANOVA ache BY array
  /METHOD=SSTYPE(3)
  /INTERCEPT=INCLUDE
  /POSTHOC=array(TUKEY BONFERRONI)
  /CRITERIA=ALPHA(0.05)
  /DESIGN=array.
```

Univariate Analysis of Variance

Notes

Output Created		20-Ara-2013 19:26:26
Comments		
Input	Data	D:\Users\aycaergul-bcc\Desktop\array.sav
	Active Dataset	DataSet1
	Filter	<none>
	Weight	<none>
	Split File	<none>
	N of Rows in Working Data File	12
Missing Value Handling	Definition of Missing	User-defined missing values are treated as missing.
	Cases Used	Statistics are based on all cases with valid data for all variables in the model.
Syntax		UNIANOVA ache BY array /METHOD=SSTYPE(3) /INTERCEPT=INCLUDE /POSTHOC=array(TUKEY BONFERRONI) /CRITERIA=ALPHA(0.05) /DESIGN=array.
Resources	Processor Time	00 00:00:00,015
	Elapsed Time	00 00:00:00,016

```
[DataSet1] D:\Users\aycaergul-bcc\Desktop\array.sav
```

Between-Subjects Factors

		Value Label	N
array	1	female old	3
	2	female young	3
	3	male old	3
	4	male young	3

Tests of Between-Subjects Effects

Dependent Variable:ache

Source	Type III Sum of Squares	df	Mean Square	F	Sig.
Corrected Model	2,221[a]	3	,740	14,192	,001
Intercept	611,426	1	611,426	11723,146	,000
array	2,221	3	,740	14,192	,001
Error	,417	8	,052		
Total	614,064	12			
Corrected Total	2,638	11			

a. R Squared = ,842 (Adjusted R Squared = ,783)

Post Hoc Tests

array

Multiple Comparisons

Dependent Variable:ache

	(I) array	(J) array	Mean Difference (I-J)	Std. Error	Sig.
Tukey HSD	female old	female young	-,756920*	,1864680	,015
		male old	-,444556	,1864680	,158
		male young	-1,175810*	,1864680	,001
	female young	female old	,756920*	,1864680	,015
		male old	,312365	,1864680	,394
		male young	-,418890	,1864680	,190
	male old	female old	,444556	,1864680	,158
		female young	-,312365	,1864680	,394
		male young	-,731254*	,1864680	,019
	male young	female old	1,175810*	,1864680	,001
		female young	,418890	,1864680	,190
		male old	,731254*	,1864680	,019
Bonferroni	female old	female young	-,756920*	,1864680	,022
		male old	-,444556	,1864680	,266
		male young	-1,175810*	,1864680	,001
	female young	female old	,756920*	,1864680	,022
		male old	,312365	,1864680	,795
		male young	-,418890	,1864680	,329
	male old	female old	,444556	,1864680	,266
		female young	-,312365	,1864680	,795
		male young	-,731254*	,1864680	,026
	male young	female old	1,175810*	,1864680	,001
		female young	,418890	,1864680	,329
		male old	,731254*	,1864680	,026

Multiple Comparisons

Dependent Variable:ache

	(I) array	(J) array	95% Confidence Interval Lower Bound	Upper Bound
Tukey HSD	female old	female young	-1,354056	-,159784
		male old	-1,041692	,152580
		male young	-1,772946	-,578674
	female young	female old	,159784	1,354056
		male old	-,284771	,909501
		male young	-1,016026	,178246
	male old	female old	-,152580	1,041692
		female young	-,909501	,284771
		male young	-1,328390	-,134118
	male young	female old	,578674	1,772946
		female young	-,178246	1,016026
		male old	,134118	1,328390
Bonferroni	female old	female young	-1,405620	-,108221
		male old	-1,093255	,204144
		male young	-1,824510	-,527110
	female young	female old	,108221	1,405620
		male old	-,336335	,961064
		male young	-1,067589	,229810
	male old	female old	-,204144	1,093255
		female young	-,961064	,336335
		male young	-1,379954	-,082555
	male young	female old	,527110	1,824510
		female young	-,229810	1,067589
		male old	,082555	1,379954

Based on observed means.
The error term is Mean Square(Error) = ,052.

*. The mean difference is significant at the 0,05 level.

Homogeneous Subsets

ache

	array	N	Subset 1	Subset 2	Subset 3
Tukey HSD[a,b]	female old	3	6,543757		
	male old	3	6,988313	6,988313	
	female young	3		7,300678	7,300678
	male young	3			7,719567
	Sig.		,158	,394	,190

Means for groups in homogeneous subsets are displayed.
Based on observed means.
The error term is Mean Square(Error) = ,052.

a. Uses Harmonic Mean Sample Size = 3,000.
b. Alpha = 0,05.

```
UNIANOVA ppargc1b BY array
  /METHOD=SSTYPE(3)
  /INTERCEPT=INCLUDE
  /POSTHOC=array(TUKEY BONFERRONI)
  /CRITERIA=ALPHA(0.05)
  /DESIGN=array.
```

Univariate Analysis of Variance

Notes

Output Created		20-Ara-2013 19:27:18
Comments		
Input	Data	D:\Users\aycaergul-bcc\Desktop\array.sav
	Active Dataset	DataSet1
	Filter	<none>
	Weight	<none>
	Split File	<none>
	N of Rows in Working Data File	12
Missing Value Handling	Definition of Missing	User-defined missing values are treated as missing.
	Cases Used	Statistics are based on all cases with valid data for all variables in the model.
Syntax		UNIANOVA ppargc1b BY array /METHOD=SSTYPE(3) /INTERCEPT=INCLUDE /POSTHOC=array(TUKEY BONFERRONI) /CRITERIA=ALPHA(0.05) /DESIGN=array.
Resources	Processor Time	00 00:00:00,015
	Elapsed Time	00 00:00:00,015

```
[DataSet1] D:\Users\aycaergul-bcc\Desktop\array.sav
```

Between-Subjects Factors

		Value Label	N
array	1	female old	3
	2	female young	3
	3	male old	3
	4	male young	3

Tests of Between-Subjects Effects

Dependent Variable:ppargc1b

Source	Type III Sum of Squares	df	Mean Square	F	Sig.
Corrected Model	11,040[a]	3	3,680	4,049	,050
Intercept	409,143	1	409,143	450,162	,000
array	11,040	3	3,680	4,049	,050
Error	7,271	8	,909		
Total	427,454	12			
Corrected Total	18,311	11			

a. R Squared = ,603 (Adjusted R Squared = ,454)

Post Hoc Tests

array

Multiple Comparisons

Dependent Variable:ppargc1b

	(I) array	(J) array	Mean Difference (I-J)	Std. Error	Sig.
Tukey HSD	female old	female young	-2,287842	,7784082	,072
		male old	-,249734	,7784082	,988
		male young	-1,686398	,7784082	,212
	female young	female old	2,287842	,7784082	,072
		male old	2,038108	,7784082	,114
		male young	,601444	,7784082	,865
	male old	female old	,249734	,7784082	,988
		female young	-2,038108	,7784082	,114
		male young	-1,436663	,7784082	,321
	male young	female old	1,686398	,7784082	,212
		female young	-,601444	,7784082	,865
		male old	1,436663	,7784082	,321
Bonferroni	female old	female young	-2,287842	,7784082	,112
		male old	-,249734	,7784082	1,000
		male young	-1,686398	,7784082	,373
	female young	female old	2,287842	,7784082	,112
		male old	2,038108	,7784082	,184
		male young	,601444	,7784082	1,000
	male old	female old	,249734	,7784082	1,000
		female young	-2,038108	,7784082	,184
		male young	-1,436663	,7784082	,613
	male young	female old	1,686398	,7784082	,373
		female young	-,601444	,7784082	1,000
		male old	1,436663	,7784082	,613

Multiple Comparisons

Dependent Variable:ppargc1b

	(I) array	(J) array	95% Confidence Interval Lower Bound	95% Confidence Interval Upper Bound
Tukey HSD	female old	female young	-4,780579	,204895
		male old	-2,742471	2,243003
		male young	-4,179135	,806339
	female young	female old	-,204895	4,780579
		male old	-,454629	4,530845
		male young	-1,891293	3,094181
	male old	female old	-2,243003	2,742471
		female young	-4,530845	,454629
		male young	-3,929400	1,056074
	male young	female old	-,806339	4,179135
		female young	-3,094181	1,891293
		male old	-1,056074	3,929400
Bonferroni	female old	female young	-4,995830	,420146
		male old	-2,957722	2,458254
		male young	-4,394386	1,021590
	female young	female old	-,420146	4,995830
		male old	-,669880	4,746096
		male young	-2,106544	3,309432
	male old	female old	-2,458254	2,957722
		female young	-4,746096	,669880
		male young	-4,144651	1,271325
	male young	female old	-1,021590	4,394386
		female young	-3,309432	2,106544
		male old	-1,271325	4,144651

Based on observed means.
The error term is Mean Square(Error) = ,909.

Homogeneous Subsets

ppargc1b

	array	N	Subset 1
Tukey HSD[a,b]	female old	3	4,783118
	male old	3	5,032852
	male young	3	6,469516
	female young	3	7,070960
	Sig.		,072

Means for groups in homogeneous subsets are displayed.
Based on observed means.
The error term is Mean Square(Error) = ,909.

a. Uses Harmonic Mean Sample Size = 3,000.
b. Alpha = 0,05.

```
UNIANOVA lmo4a BY array
  /METHOD=SSTYPE(3)
  /INTERCEPT=INCLUDE
  /POSTHOC=array(TUKEY BONFERRONI)
  /CRITERIA=ALPHA(0.05)
  /DESIGN=array.
```

Univariate Analysis of Variance

Notes

Output Created		20-Ara-2013 19:27:54
Comments		
Input	Data	D:\Users\aycaergul-bcc\Desktop\array.sav
	Active Dataset	DataSet1
	Filter	<none>
	Weight	<none>
	Split File	<none>
	N of Rows in Working Data File	12
Missing Value Handling	Definition of Missing	User-defined missing values are treated as missing.
	Cases Used	Statistics are based on all cases with valid data for all variables in the model.
Syntax		UNIANOVA lmo4a BY array /METHOD=SSTYPE(3) /INTERCEPT=INCLUDE /POSTHOC=array(TUKEY BONFERRONI) /CRITERIA=ALPHA(0.05) /DESIGN=array.
Resources	Processor Time	00 00:00:00,016
	Elapsed Time	00 00:00:00,017

```
[DataSet1] D:\Users\aycaergul-bcc\Desktop\array.sav
```

Between-Subjects Factors

		Value Label	N
array	1	female old	3
	2	female young	3
	3	male old	3
	4	male young	3

Tests of Between-Subjects Effects

Dependent Variable:lmo4a

Source	Type III Sum of Squares	df	Mean Square	F	Sig.
Corrected Model	1,124[a]	3	,375	3,020	,094
Intercept	837,040	1	837,040	6747,174	,000
array	1,124	3	,375	3,020	,094
Error	,992	8	,124		
Total	839,156	12			
Corrected Total	2,116	11			

a. R Squared = ,531 (Adjusted R Squared = ,355)

Post Hoc Tests

array

Multiple Comparisons

Dependent Variable:lmo4a

	(I) array	(J) array	Mean Difference (I-J)	Std. Error	Sig.
Tukey HSD	female old	female young	-,713214	,2875852	,138
		male old	,029708	,2875852	1,000
		male young	-,394488	,2875852	,548
	female young	female old	,713214	,2875852	,138
		male old	,742923	,2875852	,120
		male young	,318726	,2875852	,695
	male old	female old	-,029708	,2875852	1,000
		female young	-,742923	,2875852	,120
		male young	-,424197	,2875852	,493
	male young	female old	,394488	,2875852	,548
		female young	-,318726	,2875852	,695
		male old	,424197	,2875852	,493
Bonferroni	female old	female young	-,713214	,2875852	,229
		male old	,029708	,2875852	1,000
		male young	-,394488	,2875852	1,000
	female young	female old	,713214	,2875852	,229
		male old	,742923	,2875852	,195
		male young	,318726	,2875852	1,000
	male old	female old	-,029708	,2875852	1,000
		female young	-,742923	,2875852	,195
		male young	-,424197	,2875852	1,000
	male young	female old	,394488	,2875852	1,000
		female young	-,318726	,2875852	1,000
		male old	,424197	,2875852	1,000

Multiple Comparisons

Dependent Variable:lmo4a

	(I) array	(J) array	95% Confidence Interval Lower Bound	Upper Bound
Tukey HSD	female old	female young	-1,634163	,207735
		male old	-,891241	,950657
		male young	-1,315437	,526461
	female young	female old	-,207735	1,634163
		male old	-,178026	1,663872
		male young	-,602223	1,239675
	male old	female old	-,950657	,891241
		female young	-1,663872	,178026
		male young	-1,345146	,496752
	male young	female old	-,526461	1,315437
		female young	-1,239675	,602223
		male old	-,496752	1,345146
Bonferroni	female old	female young	-1,713688	,287260
		male old	-,970766	1,030182
		male young	-1,394962	,605986
	female young	female old	-,287260	1,713688
		male old	-,257551	1,743397
		male young	-,681748	1,319200
	male old	female old	-1,030182	,970766
		female young	-1,743397	,257551
		male young	-1,424671	,576277
	male young	female old	-,605986	1,394962
		female young	-1,319200	,681748
		male old	-,576277	1,424671

Based on observed means.
The error term is Mean Square(Error) = ,124.

Homogeneous Subsets

lmo4a

	array	N	Subset 1
Tukey HSD[a,b]	male old	3	8,052639
	female old	3	8,082347
	male young	3	8,476835
	female young	3	8,795561
	Sig.		,120

Means for groups in homogeneous subsets are displayed.
Based on observed means.
The error term is Mean Square(Error) = ,124.

a. Uses Harmonic Mean Sample Size = 3,000.
b. Alpha = 0,05.

```
UNIANOVA pvalb8 BY array
  /METHOD=SSTYPE(3)
  /INTERCEPT=INCLUDE
  /POSTHOC=array(TUKEY BONFERRONI)
  /CRITERIA=ALPHA(0.05)
  /DESIGN=array.
```

Univariate Analysis of Variance

Notes

Output Created		20-Ara-2013 19:28:39
Comments		
Input	Data	D:\Users\aycaergul-bcc\Desktop\array.sav
	Active Dataset	DataSet1
	Filter	<none>
	Weight	<none>
	Split File	<none>
	N of Rows in Working Data File	12
Missing Value Handling	Definition of Missing	User-defined missing values are treated as missing.
	Cases Used	Statistics are based on all cases with valid data for all variables in the model.
Syntax		UNIANOVA pvalb8 BY array /METHOD=SSTYPE(3) /INTERCEPT=INCLUDE /POSTHOC=array(TUKEY BONFERRONI) /CRITERIA=ALPHA(0.05) /DESIGN=array.
Resources	Processor Time	00 00:00:00,016
	Elapsed Time	00 00:00:00,015

```
[DataSet1] D:\Users\aycaergul-bcc\Desktop\array.sav
```

Between-Subjects Factors

		Value Label	N
array	1	female old	3
	2	female young	3
	3	male old	3
	4	male young	3

Tests of Between-Subjects Effects

Dependent Variable:pvalb8

Source	Type III Sum of Squares	df	Mean Square	F	Sig.
Corrected Model	13,646[a]	3	4,549	14,004	,002
Intercept	408,677	1	408,677	1258,243	,000
array	13,646	3	4,549	14,004	,002
Error	2,598	8	,325		
Total	424,921	12			
Corrected Total	16,244	11			

a. R Squared = ,840 (Adjusted R Squared = ,780)

Post Hoc Tests

array

Multiple Comparisons

Dependent Variable:pvalb8

	(I) array	(J) array	Mean Difference (I-J)	Std. Error	Sig.
Tukey HSD	female old	female young	-,672024	,4653311	,509
		male old	-,254019	,4653311	,945
		male young	-2,708198*	,4653311	,002
	female young	female old	,672024	,4653311	,509
		male old	,418005	,4653311	,806
		male young	-2,036174*	,4653311	,010
	male old	female old	,254019	,4653311	,945
		female young	-,418005	,4653311	,806
		male young	-2,454179*	,4653311	,003
	male young	female old	2,708198*	,4653311	,002
		female young	2,036174*	,4653311	,010
		male old	2,454179*	,4653311	,003
Bonferroni	female old	female young	-,672024	,4653311	1,000
		male old	-,254019	,4653311	1,000
		male young	-2,708198*	,4653311	,002
	female young	female old	,672024	,4653311	1,000
		male old	,418005	,4653311	1,000
		male young	-2,036174*	,4653311	,014
	male old	female old	,254019	,4653311	1,000
		female young	-,418005	,4653311	1,000
		male young	-2,454179*	,4653311	,005
	male young	female old	2,708198*	,4653311	,002
		female young	2,036174*	,4653311	,014
		male old	2,454179*	,4653311	,005

Multiple Comparisons

Dependent Variable:pvalb8

	(I) array	(J) array	95% Confidence Interval Lower Bound	95% Confidence Interval Upper Bound
Tukey HSD	female old	female young	-2,162178	,818130
		male old	-1,744173	1,236135
		male young	-4,198352	-1,218044
	female young	female old	-,818130	2,162178
		male old	-1,072149	1,908159
		male young	-3,526328	-,546020
	male old	female old	-1,236135	1,744173
		female young	-1,908159	1,072149
		male young	-3,944333	-,964025
	male young	female old	1,218044	4,198352
		female young	,546020	3,526328
		male old	,964025	3,944333
Bonferroni	female old	female young	-2,290855	,946807
		male old	-1,872850	1,364812
		male young	-4,327029	-1,089368
	female young	female old	-,946807	2,290855
		male old	-1,200826	2,036836
		male young	-3,655005	-,417344
	male old	female old	-1,364812	1,872850
		female young	-2,036836	1,200826
		male young	-4,073010	-,835349
	male young	female old	1,089368	4,327029
		female young	,417344	3,655005
		male old	,835349	4,073010

Based on observed means.
The error term is Mean Square(Error) = ,325.

*. The mean difference is significant at the 0,05 level.

Homogeneous Subsets

pvalb8

	array	N	Subset	
			1	2
Tukey HSD[a,b]	female old	3	4,927226	
	male old	3	5,181245	
	female young	3	5,599250	
	male young	3		7,635425
	Sig.		,509	1,000

Means for groups in homogeneous subsets are displayed.
Based on observed means.
The error term is Mean Square(Error) = ,325.

a. Uses Harmonic Mean Sample Size = 3,000.
b. Alpha = 0,05.

```
UNIANOVA smurf2 BY array
  /METHOD=SSTYPE(3)
  /INTERCEPT=INCLUDE
  /POSTHOC=array(TUKEY BONFERRONI)
  /CRITERIA=ALPHA(0.05)
  /DESIGN=array.
```

Univariate Analysis of Variance

Notes

Output Created		20-Ara-2013 19:29:18
Comments		
Input	Data	D:\Users\aycaergul-bcc\Desktop\array.sav
	Active Dataset	DataSet1
	Filter	<none>
	Weight	<none>
	Split File	<none>
	N of Rows in Working Data File	12
Missing Value Handling	Definition of Missing	User-defined missing values are treated as missing.
	Cases Used	Statistics are based on all cases with valid data for all variables in the model.
Syntax		UNIANOVA smurf2 BY array /METHOD=SSTYPE(3) /INTERCEPT=INCLUDE /POSTHOC=array(TUKEY BONFERRONI) /CRITERIA=ALPHA(0.05) /DESIGN=array.
Resources	Processor Time	00 00:00:00,000
	Elapsed Time	00 00:00:00,000

```
[DataSet1] D:\Users\aycaergul-bcc\Desktop\array.sav
```

Between-Subjects Factors

		Value Label	N
array	1	female old	3
	2	female young	3
	3	male old	3
	4	male young	3

Tests of Between-Subjects Effects

Dependent Variable:smurf2

Source	Type III Sum of Squares	df	Mean Square	F	Sig.
Corrected Model	6,557[a]	3	2,186	5,746	,021
Intercept	757,852	1	757,852	1992,342	,000
array	6,557	3	2,186	5,746	,021
Error	3,043	8	,380		
Total	767,453	12			
Corrected Total	9,600	11			

a. R Squared = ,683 (Adjusted R Squared = ,564)

Post Hoc Tests

array

Multiple Comparisons

Dependent Variable:smurf2

	(I) array	(J) array	Mean Difference (I-J)	Std. Error	Sig.
Tukey HSD	female old	female young	1,025849	,5035757	,252
		male old	-1,013018	,5035757	,260
		male young	,382495	,5035757	,870
	female young	female old	-1,025849	,5035757	,252
		male old	-2,038868*	,5035757	,016
		male young	-,643355	,5035757	,600
	male old	female old	1,013018	,5035757	,260
		female young	2,038868*	,5035757	,016
		male young	1,395513	,5035757	,092
	male young	female old	-,382495	,5035757	,870
		female young	,643355	,5035757	,600
		male old	-1,395513	,5035757	,092
Bonferroni	female old	female young	1,025849	,5035757	,456
		male old	-1,013018	,5035757	,474
		male young	,382495	,5035757	1,000
	female young	female old	-1,025849	,5035757	,456
		male old	-2,038868*	,5035757	,022
		male young	-,643355	,5035757	1,000
	male old	female old	1,013018	,5035757	,474
		female young	2,038868*	,5035757	,022
		male young	1,395513	,5035757	,146
	male young	female old	-,382495	,5035757	1,000
		female young	,643355	,5035757	1,000
		male old	-1,395513	,5035757	,146

Multiple Comparisons

Dependent Variable:smurf2

	(I) array	(J) array	95% Confidence Interval Lower Bound	Upper Bound
Tukey HSD	female old	female young	-,586777	2,638476
		male old	-2,625645	,599608
		male young	-1,230132	1,995121
	female young	female old	-2,638476	,586777
		male old	-3,651494	-,426241
		male young	-2,255981	,969272
	male old	female old	-,599608	2,625645
		female young	,426241	3,651494
		male young	-,217114	3,008140
	male young	female old	-1,995121	1,230132
		female young	-,969272	2,255981
		male old	-3,008140	,217114
Bonferroni	female old	female young	-,726030	2,777728
		male old	-2,764897	,738861
		male young	-1,369384	2,134374
	female young	female old	-2,777728	,726030
		male old	-3,790747	-,286989
		male young	-2,395234	1,108524
	male old	female old	-,738861	2,764897
		female young	,286989	3,790747
		male young	-,356366	3,147392
	male young	female old	-2,134374	1,369384
		female young	-1,108524	2,395234
		male old	-3,147392	,356366

Based on observed means.
The error term is Mean Square(Error) = ,380.

*. The mean difference is significant at the 0,05 level.

Homogeneous Subsets

smurf2

	array	N	Subset	
			1	2
Tukey HSD[a,b]	female young	3	7,019954	
	male young	3	7,663309	7,663309
	female old	3	8,045804	8,045804
	male old	3		9,058822
	Sig.		,252	,092

Means for groups in homogeneous subsets are displayed.
Based on observed means.
The error term is Mean Square(Error) = ,380.

a. Uses Harmonic Mean Sample Size = 3,000.
b. Alpha = 0,05.

Univariate analysis with post-hoc tests
qPCR results for the selected genes

```
UNIANOVA igf1 BY qPCR
  /METHOD=SSTYPE(3)
  /INTERCEPT=INCLUDE
  /POSTHOC=qPCR(TUKEY BONFERRONI)
  /PRINT=DESCRIPTIVE
  /CRITERIA=ALPHA(.05)
  /DESIGN=qPCR.
```

Univariate Analysis of Variance

Notes

Output Created		20-Ara-2013 17:33:28
Comments		
Input	Data	D:\Users\aycaergul-bcc\Desktop\pcr.sav
	Active Dataset	DataSet1
	Filter	<none>
	Weight	<none>
	Split File	<none>
	N of Rows in Working Data File	88
Missing Value Handling	Definition of Missing	User-defined missing values are treated as missing.
	Cases Used	Statistics are based on all cases with valid data for all variables in the model.
Syntax		UNIANOVA igf1 BY qPCR /METHOD=SSTYPE(3) /INTERCEPT=INCLUDE /POSTHOC=qPCR(TUKEY BONFERRONI) /PRINT=DESCRIPTIVE /CRITERIA=ALPHA(.05) /DESIGN=qPCR.
Resources	Processor Time	00 00:00:00,000
	Elapsed Time	00 00:00:00,000

```
[DataSet1] D:\Users\aycaergul-bcc\Desktop\pcr.sav
```

Between-Subjects Factors

		Value Label	N
qPCR	1	female old	3
	2	female young	3
	3	male old	3
	4	male young	3

Descriptive Statistics

Dependent Variable:igf1

qPCR	Mean	Std. Deviation	N
female old	,000303	,0002099	3
female young	,000514	,0000577	3
male old	,000813	,0005726	3
male young	,001263	,0001815	3
Total	,000724	,0004648	12

Tests of Between-Subjects Effects

Dependent Variable:igf1

Source	Type III Sum of Squares	df	Mean Square	F	Sig.
Corrected Model	1,560E-6[a]	3	5,200E-7	5,096	,029
Intercept	6,281E-6	1	6,281E-6	61,557	,000
qPCR	1,560E-6	3	5,200E-7	5,096	,029
Error	8,163E-7	8	1,020E-7		
Total	8,658E-6	12			
Corrected Total	2,376E-6	11			

a. R Squared = ,656 (Adjusted R Squared = ,528)

Post Hoc Tests

qPCR

Multiple Comparisons

Dependent Variable:igf1

	(I) qPCR	(J) qPCR	Mean Difference (I-J)	Std. Error	Sig.
Tukey HSD	female old	female young	-,000211	,0002608	,849
		male old	-,000510	,0002608	,279
		male young	-,000960*	,0002608	,026
	female young	female old	,000211	,0002608	,849
		male old	-,000299	,0002608	,673
		male young	-,000749	,0002608	,080
	male old	female old	,000510	,0002608	,279
		female young	,000299	,0002608	,673
		male young	-,000450	,0002608	,372
	male young	female old	,000960*	,0002608	,026
		female young	,000749	,0002608	,080
		male old	,000450	,0002608	,372
Bonferroni	female old	female young	-,000211	,0002608	1,000
		male old	-,000510	,0002608	,517
		male young	-,000960*	,0002608	,037
	female young	female old	,000211	,0002608	1,000
		male old	-,000299	,0002608	1,000
		male young	-,000749	,0002608	,125
	male old	female old	,000510	,0002608	,517
		female young	,000299	,0002608	1,000
		male young	-,000450	,0002608	,737
	male young	female old	,000960*	,0002608	,037
		female young	,000749	,0002608	,125
		male old	,000450	,0002608	,737

Multiple Comparisons

Dependent Variable:igf1

	(I) qPCR	(J) qPCR	95% Confidence Interval Lower Bound	Upper Bound
Tukey HSD	female old	female young	-,001046	,000624
		male old	-,001345	,000325
		male young	-,001795	-,000125
	female young	female old	-,000624	,001046
		male old	-,001134	,000536
		male young	-,001584	,000086
	male old	female old	-,000325	,001345
		female young	-,000536	,001134
		male young	-,001285	,000385
	male young	female old	,000125	,001795
		female young	-,000086	,001584
		male old	-,000385	,001285
Bonferroni	female old	female young	-,001118	,000696
		male old	-,001418	,000397
		male young	-,001868	-,000053
	female young	female old	-,000696	,001118
		male old	-,001207	,000608
		male young	-,001657	,000158
	male old	female old	-,000397	,001418
		female young	-,000608	,001207
		male young	-,001357	,000457
	male young	female old	,000053	,001868
		female young	-,000158	,001657
		male old	-,000457	,001357

Based on observed means.
The error term is Mean Square(Error) = 1,02E-007.

*. The mean difference is significant at the ,05 level.

Homogeneous Subsets

igf1

	qPCR	N	Subset 1	Subset 2
Tukey HSD [a,b]	female old	3	,000303	
	female young	3	,000514	,000514
	male old	3	,000813	,000813
	male young	3		,001263
	Sig.		,279	,080

Means for groups in homogeneous subsets are displayed.
Based on observed means.
The error term is Mean Square(Error) = 1,02E-007.

a. Uses Harmonic Mean Sample Size = 3,000.
b. Alpha = ,05.

```
UNIANOVA igf2bp3 BY qPCR
  /METHOD=SSTYPE(3)
  /INTERCEPT=INCLUDE
  /POSTHOC=qPCR(TUKEY BONFERRONI)
  /PRINT=DESCRIPTIVE
  /CRITERIA=ALPHA(.05)
  /DESIGN=qPCR.
```

Univariate Analysis of Variance

Notes

Output Created		20-Ara-2013 17:33:40
Comments		
Input	Data	D:\Users\aycaergul-bcc\Desktop\pcr.sav
	Active Dataset	DataSet1
	Filter	<none>
	Weight	<none>
	Split File	<none>
	N of Rows in Working Data File	88
Missing Value Handling	Definition of Missing	User-defined missing values are treated as missing.
	Cases Used	Statistics are based on all cases with valid data for all variables in the model.
Syntax		UNIANOVA igf2bp3 BY qPCR /METHOD=SSTYPE(3) /INTERCEPT=INCLUDE /POSTHOC=qPCR(TUKEY BONFERRONI) /PRINT=DESCRIPTIVE /CRITERIA=ALPHA(.05) /DESIGN=qPCR.
Resources	Processor Time	00 00:00:00,015
	Elapsed Time	00 00:00:00,015

[DataSet1] D:\Users\aycaergul-bcc\Desktop\pcr.sav

Between-Subjects Factors

		Value Label	N
qPCR	1	female old	3
	2	female young	3
	3	male old	3
	4	male young	3

Descriptive Statistics

Dependent Variable:igf2bp3

qPCR	Mean	Std. Deviation	N
female old	,00009042	,000082871	3
female young	,00032100	,000020056	3
male old	,00008698	,000031254	3
male young	,00020500	,000005568	3
Total	,00017585	,000107829	12

Tests of Between-Subjects Effects

Dependent Variable:igf2bp3

Source	Type III Sum of Squares	df	Mean Square	F	Sig.
Corrected Model	1,113E-7[a]	3	3,711E-8	17,935	,001
Intercept	3,711E-7	1	3,711E-7	179,317	,000
qPCR	1,113E-7	3	3,711E-8	17,935	,001
Error	1,656E-8	8	2,069E-9		
Total	4,990E-7	12			
Corrected Total	1,279E-7	11			

a. R Squared = ,871 (Adjusted R Squared = ,822)

Post Hoc Tests

qPCR

Multiple Comparisons

Dependent Variable:igf2bp3

	(I) qPCR	(J) qPCR	Mean Difference (I-J)	Std. Error	Sig.
Tukey HSD	female old	female young	-,00023058*	,000037143	,001
		male old	,00000343	,000037143	1,000
		male young	-,00011458	,000037143	,059
	female young	female old	,00023058*	,000037143	,001
		male old	,00023402*	,000037143	,001
		male young	,00011600	,000037143	,056
	male old	female old	-,00000343	,000037143	1,000
		female young	-,00023402*	,000037143	,001
		male young	-,00011802	,000037143	,052
	male young	female old	,00011458	,000037143	,059
		female young	-,00011600	,000037143	,056
		male old	,00011802	,000037143	,052
Bonferroni	female old	female young	-,00023058*	,000037143	,002
		male old	,00000343	,000037143	1,000
		male young	-,00011458	,000037143	,090
	female young	female old	,00023058*	,000037143	,002
		male old	,00023402*	,000037143	,001
		male young	,00011600	,000037143	,085
	male old	female old	-,00000343	,000037143	1,000
		female young	-,00023402*	,000037143	,001
		male young	-,00011802	,000037143	,078
	male young	female old	,00011458	,000037143	,090
		female young	-,00011600	,000037143	,085
		male old	,00011802	,000037143	,078

Multiple Comparisons

Dependent Variable:igf2bp3

	(I) qPCR	(J) qPCR	95% Confidence Interval Lower Bound	95% Confidence Interval Upper Bound
Tukey HSD	female old	female young	-,00034953	-,00011164
		male old	-,00011551	,00012238
		male young	-,00023353	,00000436
	female young	female old	,00011164	,00034953
		male old	,00011507	,00035296
		male young	-,00000294	,00023494
	male old	female old	-,00012238	,00011551
		female young	-,00035296	-,00011507
		male young	-,00023696	,00000093
	male young	female old	-,00000436	,00023353
		female young	-,00023494	,00000294
		male old	-,00000093	,00023696
Bonferroni	female old	female young	-,00035980	-,00010137
		male old	-,00012578	,00013265
		male young	-,00024380	,00001463
	female young	female old	,00010137	,00035980
		male old	,00010480	,00036323
		male young	-,00001322	,00024522
	male old	female old	-,00013265	,00012578
		female young	-,00036323	-,00010480
		male young	-,00024723	,00001120
	male young	female old	-,00001463	,00024380
		female young	-,00024522	,00001322
		male old	-,00001120	,00024723

Based on observed means.
The error term is Mean Square(Error) = 2,07E-009.

*. The mean difference is significant at the ,05 level.

Homogeneous Subsets

igf2bp3

	qPCR	N	Subset 1	Subset 2
Tukey HSD[a,b]	male old	3	,00008698	
	female old	3	,00009042	
	male young	3	,00020500	,00020500
	female young	3		,00032100
	Sig.		,052	,056

Means for groups in homogeneous subsets are displayed.
Based on observed means.
The error term is Mean Square(Error) = 2,07E-009.

a. Uses Harmonic Mean Sample Size = 3,000.
b. Alpha = ,05.

```
UNIANOVA igfbp2a BY qPCR
  /METHOD=SSTYPE(3)
  /INTERCEPT=INCLUDE
  /POSTHOC=qPCR(TUKEY BONFERRONI)
  /PRINT=DESCRIPTIVE
  /CRITERIA=ALPHA(.05)
  /DESIGN=qPCR.
```

Univariate Analysis of Variance

Notes

Output Created		20-Ara-2013 17:33:58
Comments		
Input	Data	D:\Users\aycaergul-bcc\Desktop\pcr.sav
	Active Dataset	DataSet1
	Filter	<none>
	Weight	<none>
	Split File	<none>
	N of Rows in Working Data File	88
Missing Value Handling	Definition of Missing	User-defined missing values are treated as missing.
	Cases Used	Statistics are based on all cases with valid data for all variables in the model.
Syntax		UNIANOVA igfbp2a BY qPCR /METHOD=SSTYPE(3) /INTERCEPT=INCLUDE /POSTHOC=qPCR(TUKEY BONFERRONI) /PRINT=DESCRIPTIVE /CRITERIA=ALPHA(.05) /DESIGN=qPCR.
Resources	Processor Time	00 00:00:00,000
	Elapsed Time	00 00:00:00,000

```
[DataSet1] D:\Users\aycaergul-bcc\Desktop\pcr.sav
```

Between-Subjects Factors

		Value Label	N
qPCR	1	female old	3
	2	female young	3
	3	male old	3
	4	male young	3

Descriptive Statistics

Dependent Variable:igfbp2a

qPCR	Mean	Std. Deviation	N
female old	,002857	,0003378	3
female young	,003183	,0004675	3
male old	,003793	,0009577	3
male young	,006845	,0041343	3
Total	,004170	,0024619	12

Tests of Between-Subjects Effects

Dependent Variable:igfbp2a

Source	Type III Sum of Squares	df	Mean Square	F	Sig.
Corrected Model	2,999E-5[a]	3	9,996E-6	2,180	,168
Intercept	,000	1	,000	45,496	,000
qPCR	2,999E-5	3	9,996E-6	2,180	,168
Error	3,668E-5	8	4,586E-6		
Total	,000	12			
Corrected Total	6,667E-5	11			

a. R Squared = ,450 (Adjusted R Squared = ,243)

Post Hoc Tests

qPCR

Multiple Comparisons

Dependent Variable:igfbp2a

	(I) qPCR	(J) qPCR	Mean Difference (I-J)	Std. Error	Sig.
Tukey HSD	female old	female young	-,000327	,0017484	,997
		male old	-,000937	,0017484	,948
		male young	-,003988	,0017484	,182
	female young	female old	,000327	,0017484	,997
		male old	-,000610	,0017484	,984
		male young	-,003662	,0017484	,233
	male old	female old	,000937	,0017484	,948
		female young	,000610	,0017484	,984
		male young	-,003052	,0017484	,363
	male young	female old	,003988	,0017484	,182
		female young	,003662	,0017484	,233
		male old	,003052	,0017484	,363
Bonferroni	female old	female young	-,000327	,0017484	1,000
		male old	-,000937	,0017484	1,000
		male young	-,003988	,0017484	,312
	female young	female old	,000327	,0017484	1,000
		male old	-,000610	,0017484	1,000
		male young	-,003662	,0017484	,417
	male old	female old	,000937	,0017484	1,000
		female young	,000610	,0017484	1,000
		male young	-,003052	,0017484	,714
	male young	female old	,003988	,0017484	,312
		female young	,003662	,0017484	,417
		male old	,003052	,0017484	,714

Multiple Comparisons

Dependent Variable:igfbp2a

	(I) qPCR	(J) qPCR	95% Confidence Interval Lower Bound	95% Confidence Interval Upper Bound
Tukey HSD	female old	female young	-,005926	,005272
		male old	-,006536	,004662
		male young	-,009587	,001611
	female young	female old	-,005272	,005926
		male old	-,006209	,004989
		male young	-,009261	,001937
	male old	female old	-,004662	,006536
		female young	-,004989	,006209
		male young	-,008651	,002547
	male young	female old	-,001611	,009587
		female young	-,001937	,009261
		male old	-,002547	,008651
Bonferroni	female old	female young	-,006409	,005756
		male old	-,007019	,005146
		male young	-,010071	,002094
	female young	female old	-,005756	,006409
		male old	-,006693	,005473
		male young	-,009744	,002421
	male old	female old	-,005146	,007019
		female young	-,005473	,006693
		male young	-,009134	,003031
	male young	female old	-,002094	,010071
		female young	-,002421	,009744
		male old	-,003031	,009134

Based on observed means.
The error term is Mean Square(Error) = 4,59E-006.

Homogeneous Subsets

igfbp2a

	qPCR	N	Subset 1
Tukey HSD[a,b]	female old	3	,002857
	female young	3	,003183
	male old	3	,003793
	male young	3	,006845
	Sig.		,182

Means for groups in homogeneous subsets are displayed.
Based on observed means.
The error term is Mean Square(Error) = 4,59E-006.

a. Uses Harmonic Mean Sample Size = 3,000.
b. Alpha = ,05.

```
UNIANOVA ache BY qPCR
  /METHOD=SSTYPE(3)
  /INTERCEPT=INCLUDE
  /POSTHOC=qPCR(TUKEY BONFERRONI)
  /PRINT=DESCRIPTIVE
  /CRITERIA=ALPHA(.05)
  /DESIGN=qPCR.
```

Univariate Analysis of Variance

Notes

Output Created		20-Ara-2013 17:34:11
Comments		
Input	Data	D:\Users\aycaergul-bcc\Desktop\pcr.sav
	Active Dataset	DataSet1
	Filter	<none>
	Weight	<none>
	Split File	<none>
	N of Rows in Working Data File	88
Missing Value Handling	Definition of Missing	User-defined missing values are treated as missing.
	Cases Used	Statistics are based on all cases with valid data for all variables in the model.
Syntax		UNIANOVA ache BY qPCR /METHOD=SSTYPE(3) /INTERCEPT=INCLUDE /POSTHOC=qPCR(TUKEY BONFERRONI) /PRINT=DESCRIPTIVE /CRITERIA=ALPHA(.05) /DESIGN=qPCR.
Resources	Processor Time	00 00:00:00,000
	Elapsed Time	00 00:00:00,000

```
[DataSet1] D:\Users\aycaergul-bcc\Desktop\pcr.sav
```

Between-Subjects Factors

		Value Label	N
qPCR	1	female old	3
	2	female young	3
	3	male old	3
	4	male young	3

Descriptive Statistics

Dependent Variable:ache

qPCR	Mean	Std. Deviation	N
female old	,004678	,0020846	3
female young	,005540	,0014556	3
male old	,006528	,0021069	3
male young	,006132	,0003672	3
Total	,005720	,0015925	12

Tests of Between-Subjects Effects

Dependent Variable:ache

Source	Type III Sum of Squares	df	Mean Square	F	Sig.
Corrected Model	5,821E-6[a]	3	1,940E-6	,703	,576
Intercept	,000	1	,000	142,256	,000
qPCR	5,821E-6	3	1,940E-6	,703	,576
Error	2,208E-5	8	2,760E-6		
Total	,000	12			
Corrected Total	2,790E-5	11			

a. R Squared = ,209 (Adjusted R Squared = -,088)

Post Hoc Tests

qPCR

Multiple Comparisons

Dependent Variable:ache

	(I) qPCR	(J) qPCR	Mean Difference (I-J)	Std. Error	Sig.
Tukey HSD	female old	female young	-,000862	,0013564	,918
		male old	-,001850	,0013564	,552
		male young	-,001453	,0013564	,715
	female young	female old	,000862	,0013564	,918
		male old	-,000988	,0013564	,883
		male young	-,000592	,0013564	,970
	male old	female old	,001850	,0013564	,552
		female young	,000988	,0013564	,883
		male young	,000397	,0013564	,991
	male young	female old	,001453	,0013564	,715
		female young	,000592	,0013564	,970
		male old	-,000397	,0013564	,991
Bonferroni	female old	female young	-,000862	,0013564	1,000
		male old	-,001850	,0013564	1,000
		male young	-,001453	,0013564	1,000
	female young	female old	,000862	,0013564	1,000
		male old	-,000988	,0013564	1,000
		male young	-,000592	,0013564	1,000
	male old	female old	,001850	,0013564	1,000
		female young	,000988	,0013564	1,000
		male young	,000397	,0013564	1,000
	male young	female old	,001453	,0013564	1,000
		female young	,000592	,0013564	1,000
		male old	-,000397	,0013564	1,000

Multiple Comparisons

Dependent Variable:ache

	(I) qPCR	(J) qPCR	95% Confidence Interval Lower Bound	95% Confidence Interval Upper Bound
Tukey HSD	female old	female young	-,005205	,003482
		male old	-,006194	,002494
		male young	-,005797	,002890
	female young	female old	-,003482	,005205
		male old	-,005332	,003355
		male young	-,004935	,003752
	male old	female old	-,002494	,006194
		female young	-,003355	,005332
		male young	-,003947	,004740
	male young	female old	-,002890	,005797
		female young	-,003752	,004935
		male old	-,004740	,003947
Bonferroni	female old	female young	-,005580	,003857
		male old	-,006569	,002869
		male young	-,006172	,003265
	female young	female old	-,003857	,005580
		male old	-,005707	,003730
		male young	-,005310	,004127
	male old	female old	-,002869	,006569
		female young	-,003730	,005707
		male young	-,004322	,005115
	male young	female old	-,003265	,006172
		female young	-,004127	,005310
		male old	-,005115	,004322

Based on observed means.
The error term is Mean Square(Error) = 2,76E-006.

Homogeneous Subsets

ache

	qPCR	N	Subset 1
Tukey HSD[a,b]	female old	3	,004678
	female young	3	,005540
	male young	3	,006132
	male old	3	,006528
	Sig.		,552

Means for groups in homogeneous subsets are displayed.
Based on observed means.
The error term is Mean Square(Error) = 2,76E-006.

a. Uses Harmonic Mean Sample Size = 3,000.
b. Alpha = ,05.

```
UNIANOVA ppargc1b BY qPCR
  /METHOD=SSTYPE(3)
  /INTERCEPT=INCLUDE
  /POSTHOC=qPCR(TUKEY BONFERRONI)
  /PRINT=DESCRIPTIVE
  /CRITERIA=ALPHA(.05)
  /DESIGN=qPCR.
```

Univariate Analysis of Variance

Notes

Output Created		20-Ara-2013 17:34:27
Comments		
Input	Data	D:\Users\aycaergul-bcc\Desktop\pcr.sav
	Active Dataset	DataSet1
	Filter	<none>
	Weight	<none>
	Split File	<none>
	N of Rows in Working Data File	88
Missing Value Handling	Definition of Missing	User-defined missing values are treated as missing.
	Cases Used	Statistics are based on all cases with valid data for all variables in the model.
Syntax		UNIANOVA ppargc1b BY qPCR /METHOD=SSTYPE(3) /INTERCEPT=INCLUDE /POSTHOC=qPCR(TUKEY BONFERRONI) /PRINT=DESCRIPTIVE /CRITERIA=ALPHA(.05) /DESIGN=qPCR.
Resources	Processor Time	00 00:00:00,000
	Elapsed Time	00 00:00:00,000

[DataSet1] D:\Users\aycaergul-bcc\Desktop\pcr.sav

Between-Subjects Factors

		Value Label	N
qPCR	1	female old	3
	2	female young	3
	3	male old	3
	4	male young	3

Descriptive Statistics

Dependent Variable:ppargc1b

qPCR	Mean	Std. Deviation	N
female old	,002235	,0015569	3
female young	,003513	,0016938	3
male old	,003765	,0015509	3
male young	,002975	,0004778	3
Total	,003122	,0013477	12

Tests of Between-Subjects Effects

Dependent Variable:ppargc1b

Source	Type III Sum of Squares	df	Mean Square	F	Sig.
Corrected Model	4,127E-6[a]	3	1,376E-6	,694	,581
Intercept	,000	1	,000	59,024	,000
qPCR	4,127E-6	3	1,376E-6	,694	,581
Error	1,585E-5	8	1,982E-6		
Total	,000	12			
Corrected Total	1,998E-5	11			

a. R Squared = ,207 (Adjusted R Squared = -,091)

Post Hoc Tests

qPCR

Multiple Comparisons

Dependent Variable:ppargc1b

	(I) qPCR	(J) qPCR	Mean Difference (I-J)	Std. Error	Sig.
Tukey HSD	female old	female young	-,001279	,0011494	,693
		male old	-,001530	,0011494	,570
		male young	-,000740	,0011494	,915
	female young	female old	,001279	,0011494	,693
		male old	-,000252	,0011494	,996
		male young	,000538	,0011494	,964
	male old	female old	,001530	,0011494	,570
		female young	,000252	,0011494	,996
		male young	,000790	,0011494	,899
	male young	female old	,000740	,0011494	,915
		female young	-,000538	,0011494	,964
		male old	-,000790	,0011494	,899
Bonferroni	female old	female young	-,001279	,0011494	1,000
		male old	-,001530	,0011494	1,000
		male young	-,000740	,0011494	1,000
	female young	female old	,001279	,0011494	1,000
		male old	-,000252	,0011494	1,000
		male young	,000538	,0011494	1,000
	male old	female old	,001530	,0011494	1,000
		female young	,000252	,0011494	1,000
		male young	,000790	,0011494	1,000
	male young	female old	,000740	,0011494	1,000
		female young	-,000538	,0011494	1,000
		male old	-,000790	,0011494	1,000

Multiple Comparisons

Dependent Variable:ppargc1b

	(I) qPCR	(J) qPCR	95% Confidence Interval Lower Bound	95% Confidence Interval Upper Bound
Tukey HSD	female old	female young	-,004959	,002402
		male old	-,005211	,002150
		male young	-,004421	,002940
	female young	female old	-,002402	,004959
		male old	-,003932	,003429
		male young	-,003142	,004219
	male old	female old	-,002150	,005211
		female young	-,003429	,003932
		male young	-,002891	,004471
	male young	female old	-,002940	,004421
		female young	-,004219	,003142
		male old	-,004471	,002891
Bonferroni	female old	female young	-,005277	,002720
		male old	-,005529	,002468
		male young	-,004739	,003258
	female young	female old	-,002720	,005277
		male old	-,004250	,003747
		male young	-,003460	,004537
	male old	female old	-,002468	,005529
		female young	-,003747	,004250
		male young	-,003209	,004789
	male young	female old	-,003258	,004739
		female young	-,004537	,003460
		male old	-,004789	,003209

Based on observed means.
The error term is Mean Square(Error) = 1,98E-006.

Homogeneous Subsets

ppargc1b

	qPCR	N	Subset 1
Tukey HSD[a,b]	female old	3	,002235
	male young	3	,002975
	female young	3	,003513
	male old	3	,003765
	Sig.		,570

Means for groups in homogeneous subsets are displayed.
Based on observed means.
The error term is Mean Square(Error) = 1,98E-006.

a. Uses Harmonic Mean Sample Size = 3,000.
b. Alpha = ,05.

```
UNIANOVA lmo4a BY qPCR
  /METHOD=SSTYPE(3)
  /INTERCEPT=INCLUDE
  /POSTHOC=qPCR(TUKEY BONFERRONI)
  /PRINT=DESCRIPTIVE
  /CRITERIA=ALPHA(.05)
  /DESIGN=qPCR.
```

Univariate Analysis of Variance

Notes

Output Created		20-Ara-2013 17:34:38
Comments		
Input	Data	D:\Users\aycaergul-bcc\Desktop\pcr.sav
	Active Dataset	DataSet1
	Filter	<none>
	Weight	<none>
	Split File	<none>
	N of Rows in Working Data File	88
Missing Value Handling	Definition of Missing	User-defined missing values are treated as missing.
	Cases Used	Statistics are based on all cases with valid data for all variables in the model.
Syntax		UNIANOVA lmo4a BY qPCR /METHOD=SSTYPE(3) /INTERCEPT=INCLUDE /POSTHOC=qPCR(TUKEY BONFERRONI) /PRINT=DESCRIPTIVE /CRITERIA=ALPHA(.05) /DESIGN=qPCR.
Resources	Processor Time	00 00:00:00,000
	Elapsed Time	00 00:00:00,000

[DataSet1] D:\Users\aycaergul-bcc\Desktop\pcr.sav

Between-Subjects Factors

		Value Label	N
qPCR	1	female old	3
	2	female young	3
	3	male old	3
	4	male young	3

Descriptive Statistics

Dependent Variable:lmo4a

qPCR	Mean	Std. Deviation	N
female old	,000895	,0003389	3
female young	,001488	,0000785	3
male old	,001063	,0003163	3
male young	,001535	,0006502	3
Total	,001245	,0004457	12

Tests of Between-Subjects Effects

Dependent Variable:lmo4a

Source	Type III Sum of Squares	df	Mean Square	F	Sig.
Corrected Model	8,973E-7[a]	3	2,991E-7	1,858	,215
Intercept	1,860E-5	1	1,860E-5	115,561	,000
qPCR	8,973E-7	3	2,991E-7	1,858	,215
Error	1,288E-6	8	1,610E-7		
Total	2,079E-5	12			
Corrected Total	2,185E-6	11			

a. R Squared = ,411 (Adjusted R Squared = ,190)

Post Hoc Tests

qPCR

Multiple Comparisons

Dependent Variable:lmo4a

	(I) qPCR	(J) qPCR	Mean Difference (I-J)	Std. Error	Sig.
Tukey HSD	female old	female young	-,000594	,0003276	,335
		male old	-,000168	,0003276	,954
		male young	-,000640	,0003276	,280
	female young	female old	,000594	,0003276	,335
		male old	,000426	,0003276	,588
		male young	-,000046	,0003276	,999
	male old	female old	,000168	,0003276	,954
		female young	-,000426	,0003276	,588
		male young	-,000472	,0003276	,511
	male young	female old	,000640	,0003276	,280
		female young	,000046	,0003276	,999
		male old	,000472	,0003276	,511
Bonferroni	female old	female young	-,000594	,0003276	,645
		male old	-,000168	,0003276	1,000
		male young	-,000640	,0003276	,519
	female young	female old	,000594	,0003276	,645
		male old	,000426	,0003276	1,000
		male young	-,000046	,0003276	1,000
	male old	female old	,000168	,0003276	1,000
		female young	-,000426	,0003276	1,000
		male young	-,000472	,0003276	1,000
	male young	female old	,000640	,0003276	,519
		female young	,000046	,0003276	1,000
		male old	,000472	,0003276	1,000

Multiple Comparisons

Dependent Variable:lmo4a

	(I) qPCR	(J) qPCR	95% Confidence Interval Lower Bound	95% Confidence Interval Upper Bound
Tukey HSD	female old	female young	-,001643	,000455
		male old	-,001217	,000881
		male young	-,001689	,000409
	female young	female old	-,000455	,001643
		male old	-,000623	,001475
		male young	-,001095	,001003
	male old	female old	-,000881	,001217
		female young	-,001475	,000623
		male young	-,001521	,000577
	male young	female old	-,000409	,001689
		female young	-,001003	,001095
		male old	-,000577	,001521
Bonferroni	female old	female young	-,001733	,000546
		male old	-,001308	,000972
		male young	-,001780	,000500
	female young	female old	-,000546	,001733
		male old	-,000714	,001565
		male young	-,001186	,001093
	male old	female old	-,000972	,001308
		female young	-,001565	,000714
		male young	-,001612	,000668
	male young	female old	-,000500	,001780
		female young	-,001093	,001186
		male old	-,000668	,001612

Based on observed means.
The error term is Mean Square(Error) = 1,61E-007.

Homogeneous Subsets

lmo4a

	qPCR	N	Subset 1
Tukey HSD[a,b]	female old	3	,000895
	male old	3	,001063
	female young	3	,001488
	male young	3	,001535
	Sig.		,280

Means for groups in homogeneous subsets are displayed.
Based on observed means.
The error term is Mean Square(Error) = 1,61E-007.

a. Uses Harmonic Mean Sample Size = 3,000.
b. Alpha = ,05.

```
UNIANOVA pvalb8 BY qPCR
  /METHOD=SSTYPE(3)
  /INTERCEPT=INCLUDE
  /POSTHOC=qPCR(TUKEY BONFERRONI)
  /PRINT=DESCRIPTIVE
  /CRITERIA=ALPHA(.05)
  /DESIGN=qPCR.
```

Univariate Analysis of Variance

Notes

Output Created		20-Ara-2013 17:34:50
Comments		
Input	Data	D:\Users\aycaergul-bcc\Desktop\pcr.sav
	Active Dataset	DataSet1
	Filter	<none>
	Weight	<none>
	Split File	<none>
	N of Rows in Working Data File	88
Missing Value Handling	Definition of Missing	User-defined missing values are treated as missing.
	Cases Used	Statistics are based on all cases with valid data for all variables in the model.
Syntax		UNIANOVA pvalb8 BY qPCR /METHOD=SSTYPE(3) /INTERCEPT=INCLUDE /POSTHOC=qPCR(TUKEY BONFERRONI) /PRINT=DESCRIPTIVE /CRITERIA=ALPHA(.05) /DESIGN=qPCR.
Resources	Processor Time	00 00:00:00,000
	Elapsed Time	00 00:00:00,000

[DataSet1] D:\Users\aycaergul-bcc\Desktop\pcr.sav

Between-Subjects Factors

		Value Label	N
qPCR	1	female old	3
	2	female young	3
	3	male old	3
	4	male young	3

Descriptive Statistics

Dependent Variable:pvalb8

qPCR	Mean	Std. Deviation	N
female old	,000041	,0000254	3
female young	,000080	,0000064	3
male old	,000085	,0000823	3
male young	,000523	,0000672	3
Total	,000182	,0002116	12

Tests of Between-Subjects Effects

Dependent Variable:pvalb8

Source	Type III Sum of Squares	df	Mean Square	F	Sig.
Corrected Model	4,686E-7[a]	3	1,562E-7	52,134	,000
Intercept	3,988E-7	1	3,988E-7	133,095	,000
qPCR	4,686E-7	3	1,562E-7	52,134	,000
Error	2,397E-8	8	2,996E-9		
Total	8,914E-7	12			
Corrected Total	4,926E-7	11			

a. R Squared = ,951 (Adjusted R Squared = ,933)

Post Hoc Tests

qPCR

Multiple Comparisons

Dependent Variable:pvalb8

	(I) qPCR	(J) qPCR	Mean Difference (I-J)	Std. Error	Sig.
Tukey HSD	female old	female young	-,000039	,0000447	,820
		male old	-,000044	,0000447	,759
		male young	-,000482*	,0000447	,000
	female young	female old	,000039	,0000447	,820
		male old	-,000005	,0000447	,999
		male young	-,000444*	,0000447	,000
	male old	female old	,000044	,0000447	,759
		female young	,000005	,0000447	,999
		male young	-,000438*	,0000447	,000
	male young	female old	,000482*	,0000447	,000
		female young	,000444*	,0000447	,000
		male old	,000438*	,0000447	,000
Bonferroni	female old	female young	-,000039	,0000447	1,000
		male old	-,000044	,0000447	1,000
		male young	-,000482*	,0000447	,000
	female young	female old	,000039	,0000447	1,000
		male old	-,000005	,0000447	1,000
		male young	-,000444*	,0000447	,000
	male old	female old	,000044	,0000447	1,000
		female young	,000005	,0000447	1,000
		male young	-,000438*	,0000447	,000
	male young	female old	,000482*	,0000447	,000
		female young	,000444*	,0000447	,000
		male old	,000438*	,0000447	,000

Multiple Comparisons

Dependent Variable:pvalb8

	(I) qPCR	(J) qPCR	95% Confidence Interval Lower Bound	Upper Bound
Tukey HSD	female old	female young	-,000182	,000104
		male old	-,000187	,000099
		male young	-,000625	-,000339
	female young	female old	-,000104	,000182
		male old	-,000149	,000138
		male young	-,000587	-,000300
	male old	female old	-,000099	,000187
		female young	-,000138	,000149
		male young	-,000581	-,000295
	male young	female old	,000339	,000625
		female young	,000300	,000587
		male old	,000295	,000581
Bonferroni	female old	female young	-,000194	,000117
		male old	-,000200	,000111
		male young	-,000638	-,000327
	female young	female old	-,000117	,000194
		male old	-,000161	,000150
		male young	-,000599	-,000288
	male old	female old	-,000111	,000200
		female young	-,000150	,000161
		male young	-,000594	-,000283
	male young	female old	,000327	,000638
		female young	,000288	,000599
		male old	,000283	,000594

Based on observed means.
The error term is Mean Square(Error) = 3,00E-009.

*. The mean difference is significant at the ,05 level.

Homogeneous Subsets

pvalb8

	qPCR	N	Subset 1	Subset 2
Tukey HSD[a,b]	female old	3	,000041	
	female young	3	,000080	
	male old	3	,000085	
	male young	3		,000523
	Sig.		,759	1,000

Means for groups in homogeneous subsets are displayed.
Based on observed means.
The error term is Mean Square(Error) = 3,00E-009.

a. Uses Harmonic Mean Sample Size = 3,000.
b. Alpha = ,05.

```
UNIANOVA smurf2 BY qPCR
  /METHOD=SSTYPE(3)
  /INTERCEPT=INCLUDE
  /POSTHOC=qPCR(TUKEY BONFERRONI)
  /PRINT=DESCRIPTIVE
  /CRITERIA=ALPHA(.05)
  /DESIGN=qPCR.
```

Univariate Analysis of Variance

Notes

Output Created		20-Ara-2013 17:35:06
Comments		
Input	Data	D:\Users\aycaergul-bcc\Desktop\pcr.sav
	Active Dataset	DataSet1
	Filter	<none>
	Weight	<none>
	Split File	<none>
	N of Rows in Working Data File	88
Missing Value Handling	Definition of Missing	User-defined missing values are treated as missing.
	Cases Used	Statistics are based on all cases with valid data for all variables in the model.
Syntax		UNIANOVA smurf2 BY qPCR /METHOD=SSTYPE(3) /INTERCEPT=INCLUDE /POSTHOC=qPCR(TUKEY BONFERRONI) /PRINT=DESCRIPTIVE /CRITERIA=ALPHA(.05) /DESIGN=qPCR.
Resources	Processor Time	00 00:00:00,000
	Elapsed Time	00 00:00:00,000

```
[DataSet1] D:\Users\aycaergul-bcc\Desktop\pcr.sav
```

Between-Subjects Factors

		Value Label	N
qPCR	1	female old	3
	2	female young	3
	3	male old	3
	4	male young	3

Descriptive Statistics

Dependent Variable:smurf2

qPCR	Mean	Std. Deviation	N
female old	,000529	,0006905	3
female young	,000058	,0000157	3
male old	,001787	,0005526	3
male young	,000309	,0004339	3
Total	,000671	,0008122	12

Tests of Between-Subjects Effects

Dependent Variable:smurf2

Source	Type III Sum of Squares	df	Mean Square	F	Sig.
Corrected Model	5,316E-6[a]	3	1,772E-6	7,302	,011
Intercept	5,401E-6	1	5,401E-6	22,258	,002
qPCR	5,316E-6	3	1,772E-6	7,302	,011
Error	1,941E-6	8	2,426E-7		
Total	1,266E-5	12			
Corrected Total	7,257E-6	11			

a. R Squared = ,733 (Adjusted R Squared = ,632)

Post Hoc Tests

qPCR

Multiple Comparisons

Dependent Variable:smurf2

	(I) qPCR	(J) qPCR	Mean Difference (I-J)	Std. Error	Sig.
Tukey HSD	female old	female young	,000471	,0004022	,660
		male old	-,001258	,0004022	,056
		male young	,000220	,0004022	,945
	female young	female old	-,000471	,0004022	,660
		male old	-,001729*	,0004022	,011
		male young	-,000251	,0004022	,922
	male old	female old	,001258	,0004022	,056
		female young	,001729*	,0004022	,011
		male young	,001478*	,0004022	,026
	male young	female old	-,000220	,0004022	,945
		female young	,000251	,0004022	,922
		male old	-,001478*	,0004022	,026
Bonferroni	female old	female young	,000471	,0004022	1,000
		male old	-,001258	,0004022	,084
		male young	,000220	,0004022	1,000
	female young	female old	-,000471	,0004022	1,000
		male old	-,001729*	,0004022	,016
		male young	-,000251	,0004022	1,000
	male old	female old	,001258	,0004022	,084
		female young	,001729*	,0004022	,016
		male young	,001478*	,0004022	,038
	male young	female old	-,000220	,0004022	1,000
		female young	,000251	,0004022	1,000
		male old	-,001478*	,0004022	,038

Multiple Comparisons

Dependent Variable:smurf2

	(I) qPCR	(J) qPCR	95% Confidence Interval Lower Bound	95% Confidence Interval Upper Bound
Tukey HSD	female old	female young	-,000817	,001759
		male old	-,002546	,000030
		male young	-,001068	,001508
	female young	female old	-,001759	,000817
		male old	-,003017	-,000441
		male young	-,001539	,001037
	male old	female old	-,000030	,002546
		female young	,000441	,003017
		male young	,000190	,002766
	male young	female old	-,001508	,001068
		female young	-,001037	,001539
		male old	-,002766	-,000190
Bonferroni	female old	female young	-,000928	,001870
		male old	-,002657	,000141
		male young	-,001179	,001619
	female young	female old	-,001870	,000928
		male old	-,003128	-,000329
		male young	-,001650	,001149
	male old	female old	-,000141	,002657
		female young	,000329	,003128
		male young	,000079	,002877
	male young	female old	-,001619	,001179
		female young	-,001149	,001650
		male old	-,002877	-,000079

Based on observed means.
The error term is Mean Square(Error) = 2,43E-007.

*. The mean difference is significant at the ,05 level.

Homogeneous Subsets

smurf2

	qPCR	N	Subset 1	Subset 2
Tukey HSD[a,b]	female young	3	,000058	
	male young	3	,000309	
	female old	3	,000529	,000529
	male old	3		,001787
	Sig.		,660	,056

Means for groups in homogeneous subsets are displayed.
Based on observed means.
The error term is Mean Square(Error) = 2,43E-007.

a. Uses Harmonic Mean Sample Size = 3,000.
b. Alpha = ,05.
