# Supplementary material for: Gene expression changes in aging Zebrafish (Danio rerio) brains are sexually dimorphic
Source: BMC Neurosci. 2014 Feb 18;15:29. doi: 10.1186/1471-2202-15-29 (PMC3937001; doi:10.1186/1471-2202-15-29)
Supplement: Additional file 7 — Genes selected for real-Time PCR experiments along with their corresponding RefSeq ID numbers used for microarray experiments and for primer design. Primer sequences are given for each gene, left primer (top) and right primer (bottom). Amplicon lengths are given as nucleotide numbers and melting temperatures used for PCR are provided. UPL; universal probe library, Tm; melting temperature, nt; nucleotides. [file 1471-2202-15-29-S7.pdf]

Gene symbol	Transcript ID from microarray	Transcript ID from UPL	Primer Sequence	Amplicon length, Tm
igf2bp3	NM_131491	NM_131491.2	caggccacttctatgcaagc<br>ccgcctcacctgagagataa	68 nt, 60°C
ache	NM_131846	NM_131846.1	cacagttctgtgctctgtgga<br>ttccactgtcgctcaacatc	85 nt, 60°C
igf1	NM_131825	NM_131825.2	gggcattggtgtgatgtctt<br>ccagtgagagggtgtgggta	67 nt, 60°C
pvalb8	NM_182937	NM_182937.2	tctatcctttccgctgatgc<br>gaactttttggggctgaatg	78 nt, 59°C
ppargc1b	XM_002664393	Ensembl *	caatgagcccagagacagaag<br>aggcacattagggggtgtc	68 nt, 60°C
smurf2	NM_001114426	NM_0011144<br>26.1	acttcctgcacacacagacg<br>ggacccaactcctcacagtt	91 nt, 59°C
lmo4a	NM_177984	NM_177984.1	gccactttatcactgtttgatacct<br>ccacacgactgttcaccatc	111 nt, 60°C
igfbp2a	NM_131458	NM_131458.1	gaccctaaagcaccacatgc<br>gagatcctttccagcacctg	71 nt, 60°C
beta actin	NM_131031	NM_181601.3	gcctgacggacaggtcat<br>accgcaagattccataccc	94 nt, 60°C

*: ENSDART00000079080.5|ENSDART00000079080.5
